# Current treatments after spinal cord injury: Cell engineering, tissue engineering, and combined therapies

**DOI:** 10.1002/SMMD.20220017

**Published:** 2022-12-26

**Authors:** Yingbo Shen, Xinyue Cao, Minhui Lu, Hongcheng Gu, Minli Li, David A. Posner

**Affiliations:** ^1^ State Key Laboratory of Bioelectronics School of Biological Science and Medical Engineering Southeast University Nanjing China; ^2^ Molecular Immunity Unit Cambridge Institute of Therapeutic Immunology and Infectious Diseases Department of Medicine University of Cambridge Cambridge UK

**Keywords:** biomaterials, drug delivery, spinal cord injury, stem cells, tissue engineering

## Abstract

Both traumatic and non‐traumatic spinal cord injuries (SCIs) can be categorized as damages done to our central nervous system (CNS). The patients' physical and mental health may suffer greatly because of traumatic SCI. With the widespread use of motor vehicles and increasingly aged population, the occurrence of SCI is more frequent than before, creating a considerable burden to global public health. The regeneration process of the spinal cord is hampered by a series of events that occur following SCI like edema, hemorrhage, formation of cystic cavities, and ischemia. An effective strategy for the treatment of SCI and functional recovery still has not been discovered; however, recent advances have been made in bioengineering fields that therapies based on cells, biomaterials, and biomolecules have proved effective in the repair of the spinal cord. In the light of worldwide importance of treatments for SCI, this article aims to provide a review of recent advances by first introducing the physiology, etiology, epidemiology, and mechanisms of SCI. We then put emphasis on the widely used clinical treatments and bioengineering strategies (cell‐based, biomaterial‐based, and biomolecule‐based) for the functional regeneration of the spinal cord as well as challenges faced by scientists currently. This article provides scientists and clinicians with a comprehensive outlook on the recent advances of preclinical and clinical treatments of SCI, hoping to help them find keys to the functional regeneration of SCI.

## Graphical abstract

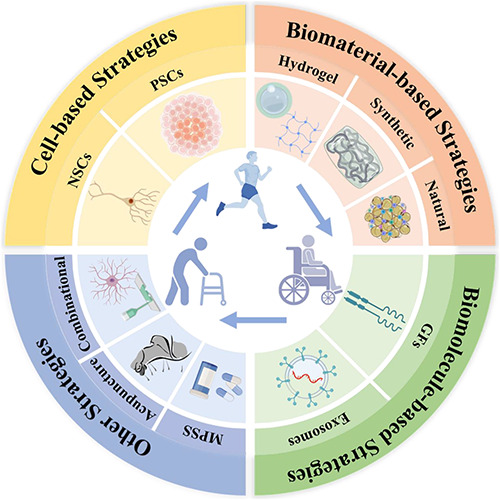

Spinal cord injury is still an intricate accident for clinicians to handle. By reviewing recent advances on cell and tissue engineering, we aim to provide an insight into the most forefront neuroprotective and neuroregenerative strategies in order to predict where the field is going in the future. We hope that this review will help accelerate the clinical transformation of SCI regeneration strategies.

1

**Key points**

- Recent advances have been made in bioengineering fields that therapies based on cells, biomaterials, and biomolecules have proved effective in the regeneration of the injured spinal cord.
- This study aims to provide an insight into the most forefront neuroprotective and neuroregenerative strategies in order to predict where the field of spinal cord injury (SCI) treatments is going in the future.
- Regardless of remarkable advances made in recent years in the area of SCI repair with various novel strategies, the successful functional regeneration of the injured spinal cord remains a complicated physiological process.

## 2 INTRODUCTION

Spinal cord injury (SCI) is referred to as the injury of our spinal cord, which, in most cases, permanently changes its function. It is also one of the most familiar impacts ensuing car accidents, severe falls, and neurological diseases.^1^ SCI is typically classified into traumatic and non‐traumatic types.^2^ Traumatic SCI is most commonly related to external physical injury such as those induced by car accidents, which cause devastating damage to our spinal cord. On the other hand, non‐traumatic SCI is usually followed by neurological chronic diseases such as tumor.^3^ Specifically, in the case of traumatic SCI, the initial injury of cells will start off an intricate secondary injury response, which can result in the death of glial cells and neural cells in a circular way. The structural organization of the spinal cord is irreversibly disrupted due to above symptoms. The fact that the spinal cord has inadequate inherent capacity of self‐healing further deepens this situation, which finally leads to eternal defects of functions in a neurological level.^2^ SCI can enforce drastic physical, mental, and social impacts on patients and their families; for example, it may cause discrimination in the work environment in people with SCI‐related diseases.^4^ The loss of independence to manage their own life and increasing death rates are also features of SCI. Moreover, the economic costs for SCI treatment and postoperative recovery are expensive,^5^ thus emphasizing the importance for us to provide effective and affordable solutions for SCI patients.

The past 40 years has witnessed an outstanding progress in this field, as considerable treatments have been translated from preclinical trials and are gradually being applied in hospitals.^6^ For example, methylprednisolone sodium succinate (MPSS) has become a widely used medicine since it was proved useful for SCI repair. Regardless of its side effects, such as increased incidence of severe pneumonia, over 85% of respondents are still willing to administer it.^7^ However, further discoveries and innovations are still needed despite previous remarkable findings for SCI treatments, for SCI to be better tackled in a pathophysiological level. For example, artificial neural scaffolds derived from synthetic or non‐synthetic polymers are structural materials that provide leading clues for cells to generate into functional tissues. They can in turn provide guidance for nerve fibers and create a stable and cell‐friendly environment for central nerve regeneration. Remarkably, various materials have been explored for tissue regeneration, including SCI repair. Not only synthetic composites such as poly‐lactic‐glycolic acid (PLGA), but natural composites like alginate as well are popular candidates for SCI regeneration.^8^ On one hand, natural polymers are sustainable materials with satisfying biocompatibility and controllable biodegradability. On the other hand, natural polymers resemble properties of extracellular matrix (ECM), making them a reliable choice for scaffolds fabrication or drug delivery vehicle.^9^ However, there still exist barriers of translating fruitful information from animal model into treatments for human.^10^ The development of hypotheses of different behaviors of cells during the recovery process of SCI and the application and combination of neural regeneration and neuroprotective strategies are fields worthy of further study.

By reviewing recent advances in cell and tissue engineering, this study aims to provide an insight into the most forefront neuroprotective and neuroregenerative strategies in order to predict where the field of SCI treatments is going in the future. We begin with the characteristics of SCI (etiology, epidemiology, and mortality). Then, mechanisms and typical symptoms are reviewed and highlighted for better understanding of following strategies. Finally, three main strategies (cell‐based, biomaterial‐based, and biomolecule‐based) will be introduced in a detailed and organized way.

## 3 CHARACTERISTICS OF SCI

### 3.1 Statistical characteristics

First, we summarize the etiology of SCI. Many reasons are accounted for SCI, including unexpected falls (regular falls and falls from a height), traffic accidents (car accidents and motor vehicle accidents), violence, sport‐related injuries and other possible causes.^11^ Among these causes, car accidents and falls are two main reasons for SCI in developed and non‐developed countries.^12^ It is also estimated that fierce impacts, like injuries related to motor vehicle and sport‐related activities, are more likely to occur in youngsters. On the other hand, mild impacts, like falls, often happen to older generations and people who often conduct work high above ground.^13^

Then, the epidemiology of SCI is concluded. The definition of incidence reflects the proportion of a new disease in a certain population during a specific time.^14^ Typically, the incidence of SCI mirrors our control level over it, while the prevalence of SCI poses a threat to our health care system and the endurance ability of our social and economic resources.^15^ Patients' demographic databases on SCI are typically grouped into two categories: nontraumatic and traumatic, reflecting the distinctions between these two categories epidemiologically. In most cases, data are collected by provincial or regional databases, which make it difficult and inaccurate to draw conclusions in a global scale. What is more, data are usually collected based on the admission rate of the hospital which apparently excludes those who cannot afford to go to a medical institution, failing to reflect the authentic prevalence and incidence of SCI.^16^ We take epidemiological features of SCI in China as an example. It can be concluded from various articles that the majority of patients are married (approximate 85%).^17^ The ratio of males to females with SCI is around 5:1. Workers account for 28.6% of the patients, followed by office employees and retired people. People aged between 30 and 49 years are the high‐risk group to have SCI. Unexpected falls are the main reason for having SCI (41.3%), followed by traffic accidents (22.3%), and being struck by objects (18.6%).

Finally, the mortality of SCI is of equal importance. Despite the fact that the survival rate of SCI has increased over the past 20 years, the death rates still exceed those of parallel diseases. It can also be concluded that death rates increase with older patients and usually an injury in a higher level of the spinal cord means more severe symptoms and higher mortality rates.^18^ It is worth noting that according to a study, patients' life span and quality of life can be effectively improved through the development of modern apps,^19^ which provides us a clue on the effective methods for postoperative repair.

### 3.2 Pathophysiology characteristics

The two categories of SCI are typically traumatic and nontraumatic. We will focus on the former group (traumatic) in the following sections. An abrupt, destructive force applied on the spine which shatters or dislocates the vertebral column from its original location is typically the cause of traumatic SCI. Primary injury and secondary injury are two major parts of traumatic SCI.^20^

#### 3.2.1 Primary injury

The initial mechanical force applied on the spinal cord is referred to as the primary injury, after which irreversible damage of neural cells and spinal tissue ensues. Morphologically, four main features of primary injury are: distraction, tear, single impact with transient compression, and impact with continuous compression.^21^ What is more, the disturbance of blood–spinal cord barrier (BSCB) together with rapid ischemia is the by‐product of it. Apoptosis and necrosis in neural and glial cells thus occur, following subsequent edema and hemorrhage.^22^ These physical and cellular damages combined unavoidably start off a continuous secondary injury chain reaction, which may last weeks or months and induce severer damage in a cellular level than those of primary injuries.

#### 3.2.2 Secondary injury

Secondary injury closely follows the primary injury, which is characterized by a series of neurological deficiencies. Secondary SCI is divided into four stages in the pathophysiological level: the acute (within 48 h), subacute (48 h to 14 days), intermediate (14 days to 6 months), and chronic (more than 6 months) stages.^23^ The detailed descriptions are as follows:

First, the acute phase (<48 h) starts off simultaneously when SCI occurs. In this process, the main features may include imbalance of various ions, formation of free radicals, hyperexcitability of cells, edema, inflammation, apoptosis, and necrocytosis.^24^ What is more, the injury of the blood vessel tends to initiate serious hemorrhages. It first occurs in gray matter during the initial 30 min of injury, which subsequently spreads into the nearby white matter quickly. This damage to the white and gray matter will expose the injured site to a huge amount of inflammatory cells from the cytoplasmic matrix. This may cause more permeable BSCB, which in turn increases the number of inflammatory cells. This phenomenon further enhances the swelling of the spinal cord. Cell death is a major event in the secondary injury, which is partially due to the overwhelming accumulation of extracellular amino acids like aspartate and glutamate. Their receptors cause the cells to become over activated and toxic.^24^ Various chemical factors in SCI account for different mechanisms in cell death. SCI includes two major types of cell death, which are necrosis and apoptosis. Apoptosis is one of the most known forms of cell death, thus we will discuss it below. It is programmed and happens within hours of SCI, featuring the dependence of energy.^23^ These cells are lucky enough to survive but the damage unfortunately sets off the apoptotic pathways. Typically, extrinsic and intrinsic pathways combined activate the apoptosis. Among them, the influx of Ca^2+^ mainly sets off this process. Meanwhile, Fas‐mediated cell death attributes mostly to the mechanism of apoptosis during SCI.^25^ In the light of this, anti‐apoptotic treatments are probably keys to unlock the SCI recovery myth.

Then, during the transformation of acute to subacute stage (48 h to 14 days), the disappearance of ionic homeostasis tends to occur. Ca^2+^ dysregulation is one of the most noticeable losses of ionic homeostasis, contributing to cell death.^26^ Moreover, continuous necrosis of glia and neural cells may induce the activation of microglial cells. Along with the advent of further activated cells, such as lymphocyte and macrophage, together they will cause the infiltration of the injury site, where a vicious circle is triggered contributing to the apoptosis of even more cells. The clearance of myelin debris caused by phagocytic inflammation will induce further damages like delayed apoptosis, because by‐products such as free radicals will cause the peroxidation of substances like lipids and protein.^27^ Apart from overall impacts caused by SCI, the self‐regulatory function of the spinal blood vessels is further damaged, which will cause continuous hemorrhage lasting for days.^28^ In conclusion, the different types of neuronal death, resulting from SCI in its acute and subacute stages, are likely to induce even more serious injury than those of the primary ones, which will in turn provide us the clue to construct basic strategies for the intervention of neuroprotection. It is also worth noting that the role of astrocyte varies throughout the SCI, starting from a neuroprotective function in the first place to an inhibitory factor in the final phase.^29^

Finally, the intermediate and chronic injury phase (>14 days) mark the downside of the extensive inflammatory response, where the spinal cord injury transforms from the dynamic state to the chronic state. This stage is characterized by the degeneration of axons and the final formation of the cystic cavitations and the maturation of glial scar.^30^ Such features will make the regrowth of axons and the migration of cells even harder because of the aggregation of cystic cavities. Cystic cavitation is constructed by the accumulative death of cells which form the vacuum space.^31^ In this case, extracellular fluid and gathered macrophages together form an almost impenetrable barrier to direct the regrowth of axons and the migration of cell. Moreover, the glial scar is made of an inhibitory array of tightly convolved astrocytes, which is situated around the cystic cavities.^32^ The glial scar is composed of astrocytes and molecules such as proteoglycan.^10^ However, the glial scar does not totally serve as an inhibitory barrier, because it is responsible for the isolation of the injury site during the initial stages. Moreover, chances are that perifocal astrocytes can help support the reconstruction of the blood vessels.^33^ In conclusion, the dual role of glial scar remains to be further discussed.

### 3.3 Current clinical treatments for central nervous system (CNS) injury

“Time is spine” has been proposed as a crucial concept in the treatment of patients with SCI.^34^ Therefore, taking actions promptly after a devastating event is of extreme importance in order to secure the success of neurological operations. The simple fact that conducting immediate treatments after SCI can potentially accelerate the recovery process and increase patients' quality of life index.^10^ Among various treatments, the use of MPSS and spinal decompression surgery are two major therapies.^35^

The use of MPSS is one of the most controversial topics in the treatment of SCI historically. MPSS is a synthetic glucocorticoid which has cytoplasmic receptors as its effectors.^36^ It can reduce inflammation by upregulating anti‐inflammatory factors. Its function had also been proved through clinical validation as well as animal models. However, the main dispute of this practice lies in the possibility that the administration of MPSS can trigger complications.^37^ In conclusion, we think this practice ought to be left for the doctor to decide whether to apply MPSS based on different symptoms of specific patients.

Another typical therapy is surgical decompression, which is the footstone of the treatment for traumatic SCI.^38^ The surgery aims to mitigate hemorrhage and edema by recovery of the stability of the spinal column, which can relieve the pressure enforced on the spinal cord. Failure to decompress the spinal cord is likely to cause a more severe secondary injury. In conclusion, the early action of decompressing the spinal cord at the injury site can potentially improve the symptoms of patients. What is more, Badhiwala et al. implied that timely decompression can shorten the recovery period for patients,^39^ which is crucial to reduce the pain and medical costs for patients. Nonetheless, these above therapies (surgical decompression and pharmacotherapy) cannot offer remarkable improvements in the neurological recovery of SCI. Therefore, the current situation calls for more effective approaches to regenerating axon by withdrawing inhibitory factors, introducing guiding cues, delivering growth factors as well as biomaterial‐based transplant, in order to simulate the inner nerve cell environment. In this detailed review, we mainly discussed the recent breakthroughs in the SCI therapies.

## 4 CELL‐BASED STRATEGIES FOR SCI RECOVERY

Cell‐based strategy is a novel and powerful therapy which utilizes the potential of various cells to treat multiple diseases, even including those seemingly incurable before.^40^ There are basically two ways to realize this strategy.^41^ First is the exogenous way, in which scientists use exogenous, for example, neural stem cells (NSCs), pluripotent stem cells (PSC), etc., for transplantation. It has the potential to repair the injured site by providing supportive materials or biological functions to survived cells. Another way is using additional leading cues to strengthen the functions and vitality of endogenous cells. The specific effects and mechanisms of this strategy is based on cell types, the time of intervention, supplying regeneration factors, treatment objects, and types of diseases.^42^ Many studies have emerged recently on exploring the effectiveness of various cells in preclinical and translational SCI treatments.^42^ ^b^ Various kinds of cells have been studied by researchers by now, including NSCs, Oligodendrocyte precursor cells (OPCs), induced pluripotent stem cells (iPSC), human embryonic stem cells (hESC), microglia, and astrocytes.^43^ In this section, in order to review in a concise and precise way, we will concentrate on one of few most trending and promising stem cell types.

Stem cells are renowned for their neuroprotective and neuroregenerative characteristics, thus making them a promising cell type for SCI recovery. Murine models of different phases of SCI have been constructed to confirm such roles of stem cells.^44^ Thanks to the active differentiation state and the secretion of various growth factors and molecules, the introduction of exogenous cells and activation of endogenous cells prove to play a therapeutic role in cellular and molecular levels.

### 4.1 Neural stem cells

NSCs are multi‐potent cells with renewing abilities. They can replace the neurons, oligodendrocytes, and astrocytes after SCI.^45^ NSCs are found in the neural tube in embryos, and they mainly reside in the spinal cord and brain in adults.^46^ SCI can trigger the NSCs, converting them into an active state. Consequently, NSCs can proliferate and differentiate into corresponding cells and migrate into injured sites, beginning the regeneration process.^47^ Experimental studies on mice of transplanting NSCs into the injury site have proved the effective role of them. It has been confirmed that NSCs can differentiate into glial cells and neurons to facilitate the functional SCI recovery.^48^ Two mechanisms are constructed to explain the functional SCI recovery after transplanting NSCs^49^: (1) the myelination of axon through NSCs‐derived oligodendrocytes and (2) the restoration of signal transduction through NSCs‐derived neurons.

**FIGURE 1:**
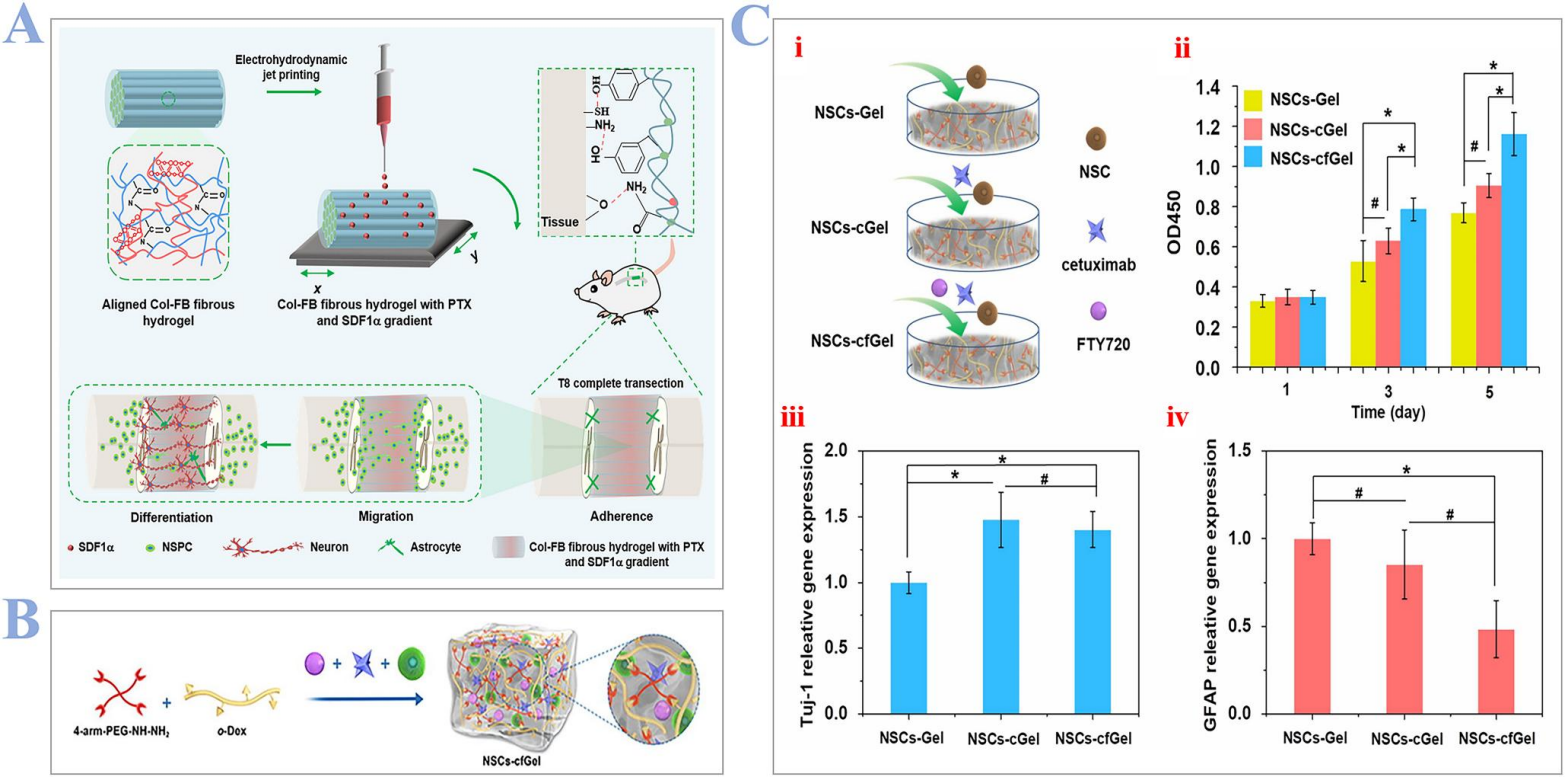
(A) Schematic illustration of collagen–fibrin fibrous hydrogels to improve functional recovery in SCI rats. Reproduced with permission.^50^ Copyright 2022, ACS Publications. (B) Schematic illustration of an injectable dual‐drug hydrogel encapsulated with NSCs to facilitate the regeneration of SCI. (C) (i) Schematic illustrations of three experimental groups. (ii) Optical density values of NSCs in each group. (iii) Analysis pf neural‐related genes (Tuj‐1) and (iv) genes related to astrocytes (GFAP) in each hydrogel after 7 days. Reproduced with permission.^51^ Copyright 2022, Elsevier. NSC, neural stem cells; SCI, spinal cord injury.

Distinctively, Chen et al. developed fibrous hydrogels made from aligned collagen/fibrin materials (Col‐FB). Furthermore, the introduction of factor‐1α (SDF1α) and paclitaxel (PTX) by the electrohydrodynamic jet printing method enables hydrogels with spatiotemporal delivery ability, which can recruit endogenous NSCs in situ for SCI repair (Figure 1A).^50^ On one hand, the encapsulation of SDF1α facilitates the migration of NSCs. On the other hand, the encapsulation of PTX facilitates the differentiation of NSCs. In vivo experiments confirmed that Col‐FB fibrous hydrogels decorated with SDF1α and PTX particles can promote the functional locomotor recovery of SCI rats greatly. Interestingly, Qi et al. choose to encapsulate NSCs directly into the dual‐drug enhanced hydrogels for SCI repair, as shown in Figure 1B.^51^ The hydrogel is formed through crosslinking 4‐arm‐PEG‐NHNH2 with o‐Dex by hydrazone bonds in PBS (pH = 7.4), and two drugs (cetuximab and FTY720) are added afterwards. In vitro experiments demonstrated that the incorporation of cetuximab and fingolimod (FTY720) can improve the proliferation and differentiation ability of NSCs, as shown in Figure 1C. However, considering the specific region where different cell groups belong, they are not the best option for SCI recovery.^52^

### 4.2 Pluripotent stem cells

PSCs have infinite proliferation and differentiation abilities. These two unique properties make PSCs ideal sources for cell‐based therapies for various diseases like heart failure, macular degeneration, and type 1diabetes. iPSCs and hESCs are two most used types of human PSCs.^41^

iPSCs and ESCs are capable of treating various diseases, like Parkinson's disease, cartilage defect, spinal cord injury, etc. After removing the trophectoderm cells, hESCs are collected from the inner cell colony of the blastocysts before implantation.^53^ Markers are needed to confirm the successful isolation of the hESCs after surgery and maintain the proliferation and differentiation abilities of hESCs. Alkaline phosphatase and telomerase are reported to represent the vitality,^54^ proliferative, and differential ability of hESCs. Apart from the amazing proliferation ability, hESCs demonstrate pluripotency both in vivo and in vitro. hESCs can differentiate into ectodermal cells like neural and glial cells, making them a suitable object for preclinical SCI studies.^55^ However, when it comes to clinical use, despite being a revolutionary technology, hESCs still have issues with ethical boundaries for using human embryos and the possibility of immune rejection.^56^ To overcome these problems, the successful generation protocol of iPSCs is needed by scientists more than ever to replace the clinical use of hESCs.

There is another approach to acquiring PSCs which are collected from somatic cells. Researchers hypothesized that pluripotency may be acquired in somatic cells through certain factors found in hESCs.^57^ They actually did confirm four crucial transcription factors which can induce pluripotency in mice and adult cells through viral transduction.^54^ ^a,^ ^58^ We name the derived cells as iPSCs. The transplantation of patients' iPSCs for SCI recovery is indeed practical, provided that they solve the ethical issues and immunological rejection of hESCs. However, reprogramming technology and tumorigenicity still remain seemingly intractable problems.^59^

Although various cells, including NSCs and Olfactory ensheathing cells (OECs), have been studied so far, the stem cells hold the potential to differentiate into neurons, oligodendrocytes, etc., which can facilitate the regeneration of the injured spinal cord. Generally, we name these cells as neural progenitor cells (NPCs). The differentiation of hESCs and iPSCs as discussed above are two approaches to acquire NPCs. The mechanisms for the regeneration of the spinal cord following NPCs transplantation are: (1) integration of neurons into new neural circuits through the formation of synapses with host cells and (2) remyelination of the denuded axons by producing neuroprotective and neuroregenerative agents.^60^

**FIGURE 2:**
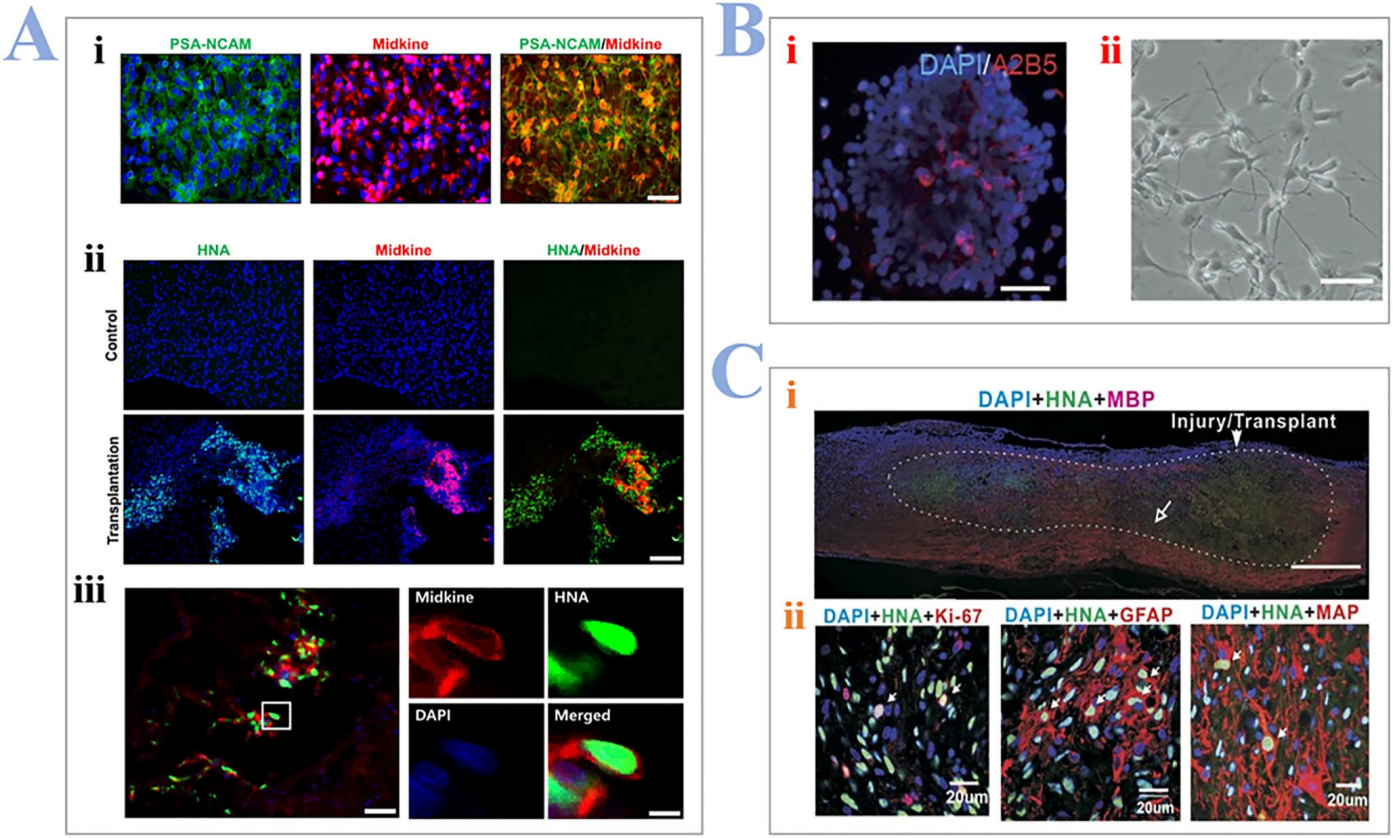
(A) Expression of midkine in human neural precursor cells (hNPCs^PSA−NCAM+^). (i) In vitro expression, scale bar: 50 μm. (ii), (iii) In vitro expression, scale bar: 100 μm (ii), 50 μm (iii, left panel) 10 μm (iii, right panel). Reproduced with permission.^61^ Copyright 2022, Springer Nature. (B) (i) A2B5‐positive cells and (ii) bright field image of OPCs. Scale bars represent 25 μm. (C) (i) Image of the lesion area illustrates the transplant site, and injury‐induced lesion cavities (the dotted white line) are transplanted with OPC‐like cells. (ii) Other types of surviving cells that are derived from the transplants (white arrows): GFAP‐positive, MAP‐positive, and Ki67‐positive. Reproduced with permission.^62^ Copyright 2017, Springer Nature. OPCs, oligodendrocyte precursor cells.

Kim et al. demonstrated the precursors with poly‐sialylated neural adhesion molecule (PSA‐NCAM), derived from hESCs can promote the functional recovery of SCI.^61^ They also detected the expression of midkine which is a crucial neurotrophic factor for regulating neural regeneration, as presented in Figure 2A. In 2017, Dae‐Sung Kim and colleagues explored the differentiation of OPCs for the purpose of treating SCI.^62^ They selected cells with A2B5‐positive marker from hPSCs‐derived tissues and used the signaling messages (sonic hedgehog, growth factor‐1, etc.) to culture the acquired cells. In this way, they achieved the rapid acquirement of OPC‐like cells as presented in Figure 2B. Furthermore, the immunostaining of grafts after eight weeks of transplantation meant that the simple introduction of OPCs is conducive for the SCI regeneration as presented in Figure 2C. Except differentiating into myelin‐forming oligodendrocytes, OPCs derived from hESCs can facilitate the survival of neural cells and extension of neurite by secreting neural factors like activin A and brain‐derived neurotrophic factor (BDNF).^63^

**FIGURE 3:**
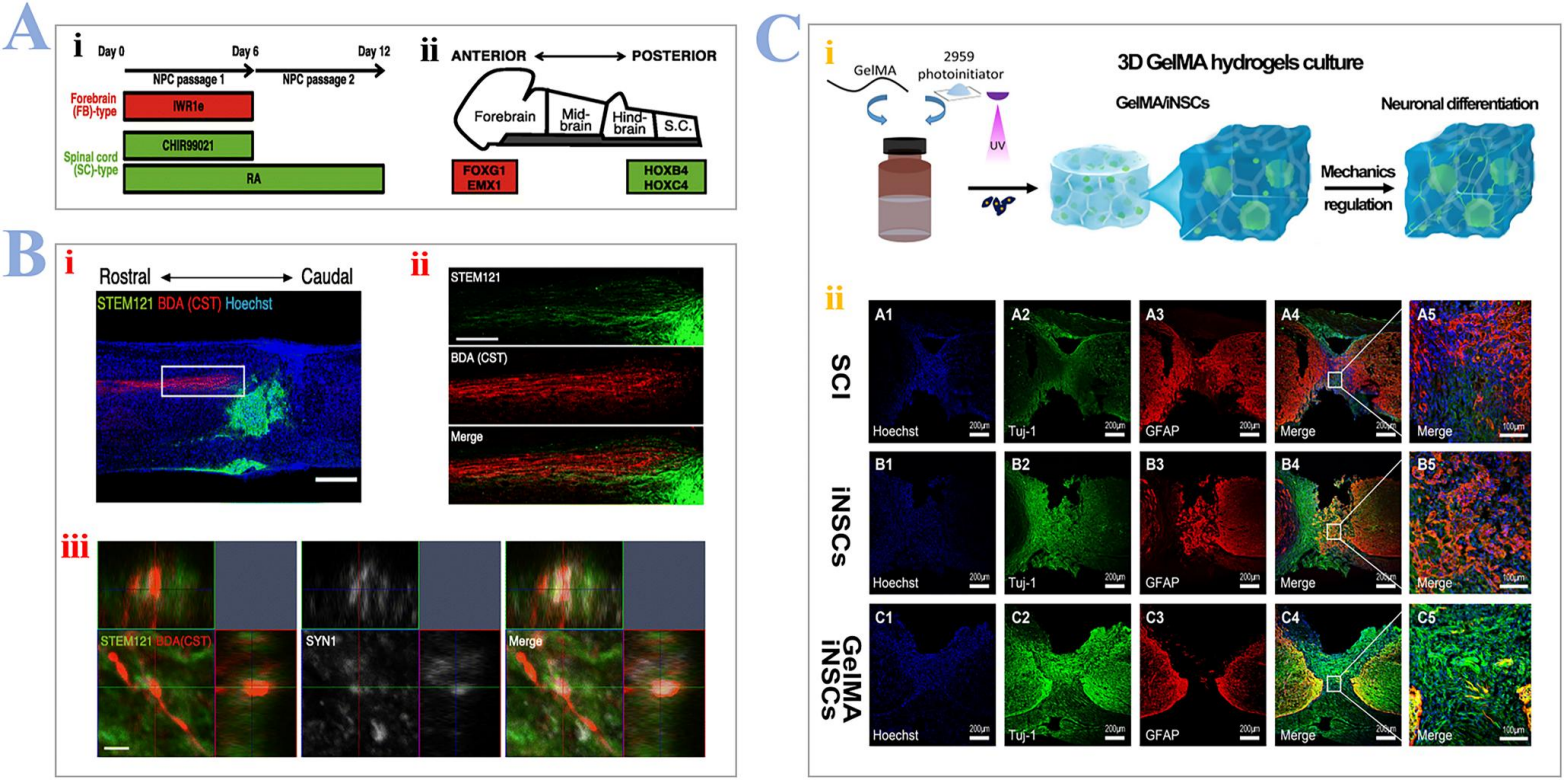
(A) (i) Schematic illustration of the culture process. Factors were supplied during the induction process of cells. (ii) In vivo expression of regional‐specified markers. (B) High (ii) and low (i) magnification image of corticospinal tract (CST) axons with STEM121‐positive SC‐type cells. Scale bar: 200 μm (ii) and 100 μm (i). (iii) Confocal images demonstrating the formation of synapse between CST axons and SC‐type transplants. The upper square is the *XZ*‐plane, and the right square is the *YZ*‐plane. Scale bar: 2 μm. Reproduced under terms of the CC‐BY license.^64^ Copyright 2020, The Authors, published by Springer Nature. (C) (i) Schematic illustrations of synthesis process of hydrogel: a mixed solution of GelMA and iNSCs cross‐linked by 2959 photoinitiator under the irradiation of UV light. (ii) A1−C5 represent immunofluorescent staining pictures of GFAP (red) and Tuj‐1 (green) at the injury site. Reproduced with permission.^65^ Copyright 2018, ACS Publications. iNSCs, iPSC‐derived NSCs.

Keita Kajikawa et al.^64^ demonstrated that the effectiveness of SCI recovery is also decided by the specific region where NPC‐derived iPSCs come from. They first regulated Wnt and RA neural transduction, which are two neural signal pathways, to acquire the wanted region‐specific NPCs (Figure 3A(i)), and separate the fore‐brain and spinal cord type (FB, SC‐type) (Figure 3A(ii)). They further used a mouse to transplant the regional specified NPCs into the lesion sites in the spinal cord, then confirmed the effectiveness of FB‐type NPCs by immunostaining and immune electron microscopy (IEM) as presented in Figure 3B. Results also suggested that transplanted cells with only spinal cord identity have the potential to promote the behavioral results after SCI. Fan et al. constructed 3D GelMA hydrogels encapsulated with iPSC‐derived NSCs (iNSCs) by photoinitiator 2959 as presented in Figure 3C(i). They further confirmed GelMA hydrogels loaded with iNSCs can promote neural differentiation and inhibit the formation of astrocytic in vivo by immunofluorescent staining of Tuj‐1 and GFAP at the lesion sites as presented in Figure 3C(ii).

Although the trend for using PSCs for SCI recovery is promising, many challenges need to be overcome to fully grasp this technology. Heterogeneity, immunogenicity, and tumorigenicity are the top issues we need to address first.^41^ Heterogeneity means PSC lineages are not identical to each other in morphology, growth rate, etc., causing a barrier for downstream applications. Immunogenicity means immune rejection may occur after transplantation. Tumorigenicity means the infinite proliferation characteristic of PSCs may result in tumors after transplantation.

### 4.3 Bone marrow stem cells and mesenchymal stem cells

**FIGURE 4:**
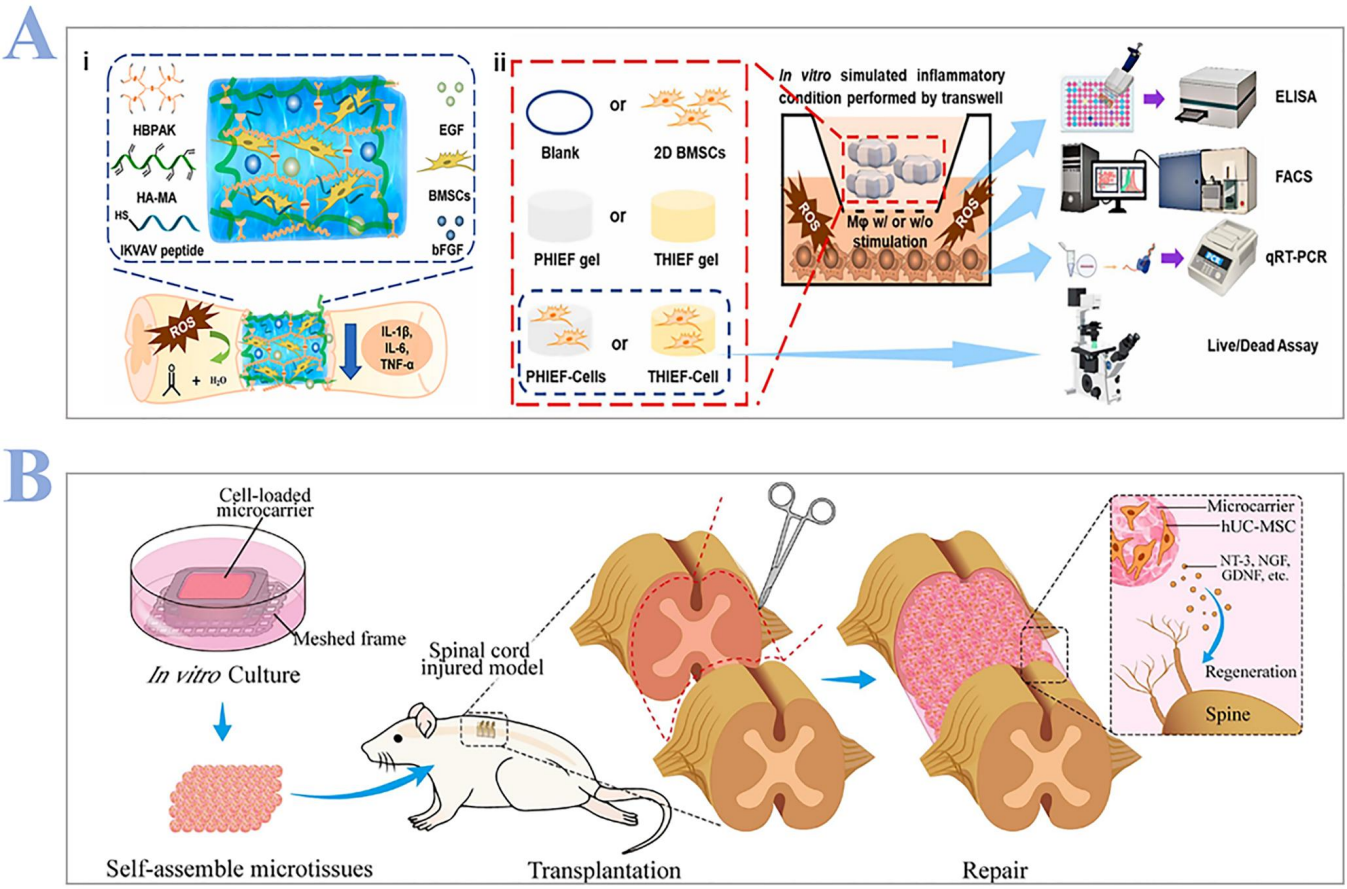
(A) (i) Schematic illustration of the BMSCs encapsulated ROS‐scavenging hydrogel for SCI recovery. After SCI, the BMSCs encapsulated ROS‐scavenging hydrogel (THIEF‐Cell) can eliminate the over‐produced ROS. (ii) Schematic illustration of the SCI model, six experimental groups at different conditions in vitro and methods to confirm the effectiveness of BMSCs encapsulated hydrogels. Reproduced with permission.^68^ Copyright 2023, The Authors, published by Elsevier. (B) Schematic illustration of how the gelatin microcarrier‐based MSCs microtissues are prepared. Reproduced with permission.^70^ Copyright 2023, Elsevier. BMSC, bone marrow stem cell; MSCs, mesenchymal stem cells; SCI, spinal cord injury.

Bone marrow stem cell (BMSC) therapy has been used to treat severe clinical inflammatory illnesses such as pancreatitis and localized cerebral ischemia. It has typical immunomodulatory properties.^66^ Due to these unique properties, BMSCs have been widely applied in clinical use. For instance, Sharma et al.^67^ reported the outcomes of the intrathecal transplantation of bone marrow‐derived mononuclear cells (BMMNCs) in pediatric patients, finding that 25% of them showed improvements in their ASIA impairment scale classification, while the other patients also displayed some neurological improvement, such as increased muscle strength, and improved bladder control. Recent research also focuses on BMSCs‐based therapy. For example, Li and coworkers^68^ created a thioketal‐containing and ROS‐scavenging hydrogel for the encapsulation of BMSCs. In this study, they first prepared the prepolymer solutions, then BMSCs were collected and resuspended at a density of 8 × 10^6^ cells per milliliter in the hydrogel prepolymer solutions when their attachment rate reached 70%–80% (Figure 4A(i)). They conducted six different experimental groups (hydrogels with or without BMSCs encapsulation, pure 2D culture of BMSCs, and pure hydrogels) to confirm that their hydrogels encapsulated with BMSCs can stimulate neurogenesis and axon regeneration by scavenging the excess ROS and reestablishing a regenerative milieu (Figure 4A(ii)).

As for mesenchymal stem cells (MSCs), in addition to their capacity for self‐renewal and multipotency, they are renowned for their paracrine trophic properties. What is more, MSCs have been used in a number of early phase clinical trials to treat SCI and results generally support the feasibility of cell transplantation administered intrathecally, intralesionally, or intravenously.^69^ Recently, Liu and coworkers^70^ produced functional tissue constructs by self‐assembly of MSC microtissues comprising of porous gelatin microcarriers (GM) and MSCs. They first seeded MSCs in GM and cultured for 3 days to form MSC‐laden GM, then functional tissue constructs were formed by self‐assembly in 3D‐printed meshed frames. Afterwards, tissue constructs were used for transplantation in a rat complete transection model of SCI (Figure 4B).

It is worth noting that cell‐based strategies alone are far from enough to realize the functional recovery of spinal cord. Therefore, novel strategies based on the integration of valid theories, proficient surgery skills along with application of medications and biomaterials need to be developed. Over the years, various approaches have been investigated to further facilitate cell‐based SCI recovery. In conclusion, apart from solving problems like ideal stem cell sources and safety of cell‐based surgeries, we must focus on exploring bioengineering technologies, that is, biomaterials/scaffold‐based and biomolecules‐based SCI treatments to revolutionize the field of SCI recovery.

Clinically, cell therapy, especially stem cell therapy, has many uncertainties to consider before being fully publicized.^67^ Cell dose, for example, is among the most important clinical variables. The differences of immune systems between patients are mainly considered for this problem. Typically, the cell dose ranging from 10^6^ to 10^10^. Furthermore, the application route is another tricky problem. Clinically, intra‐arterial, intravenous, intrathecal, and intraspinal are three major options. But each of them have their advantages and disadvantages. For example, although the intraspinal approach can usually achieve the highest level of cell engraftment but the risk of additional damage and possible infection caused by injection needles should not be underestimated. In conclusion, a further assessment in clinical research and basic experimentations are needed before cell therapy being fully publicized.

## 5 SCAFFOLDS/BIOMATERIAL‐BASED SCI STRATEGIES

Implantable scaffolds are developed to restore and reconstruct the damaged organs and functional tissues. Among them spinal cord injury is a suitable option to apply this method. Scaffolds are temporary templates for the restoration of the injury sites by promoting cell proliferation, cell differentiation, cell attachment, reconstructing nerve circuits, blood vessels, etc. Scaffolds are typically three‐dimensional (3D) permeable biomaterials with biocompatibility, which enable the transport of molecules, gases, liquids, etc.^71^ Scaffolds are designed to have minimal toxicity to cells, and to induce minimal inflammation. The structures should also have the capacity to degrade slowly and allow tissue regeneration to take its place. Three‐dimensional scaffolds can provide mechanical support at injury sites and act as a delivery system for various biomolecules like antibiotics, growth factors, drugs, etc. The scaffolds also serve as attaching points for exogenous and endogenous cells to establish new centers for regeneration. With the combination of computer‐aided design (CAD) softwares and novel bioprinting methods (3D printing), individualized scaffolds based on patients' personalized data could be constructed in macro‐, micro‐, and even nano‐scale.^72^ Researchers focused on introducing additional modification on scaffolds, such as adding extracellular matrix components (ECM proteins) and delivering biomolecules like growth factors.^73^

In conclusion, 3D scaffolds can provide physical, chemical, mechanical, biological, and geometrical cues for tissue regeneration in SCI recovery. The characteristics of biomaterials like surface energy, hydrophilicity should be carefully considered depending on specific functions and locations of scaffolds in tissue engineering.

### 5.1 Hydrogels

Hydrogels are one of the most widely used biomaterials among other scaffolds.^74^ Hydrogels consist of cross‐linked hydrophilic polymer chains. Several crosslinking methods can be applied to form gels like micro‐scale fibers^75^: photo polymerization, chemical reaction (noncovalent and covalent bonds), physical crosslinking (hydrogen bonds, electrostatic ionic force, host‐guest complexation, stereo‐complexation, etc.)^76^ solvent out, etc. Biomaterials include natural (alginate, fibrin, gelatin, etc.) and synthetic (PLGA, polylactic acid [PLA], polyethylene glycol [PEG], etc.) ones. Recent studies suggest that hydrogels are suitable candidates for SCI regeneration because they have a similar mechanical property of the spinal cord and can be easily tuned to resemble anatomical structures of the spinal cord.^74^ What is more, hydrogels can serve as a steady drug delivery system and have controllable degradation rate, vesting them properties of natural ECM. It is worth noting that “smart hydrogels” are also the focus of the field because they can respond to environment (light, temperature, pressure, etc.) accordingly.^77^

**FIGURE 5:**
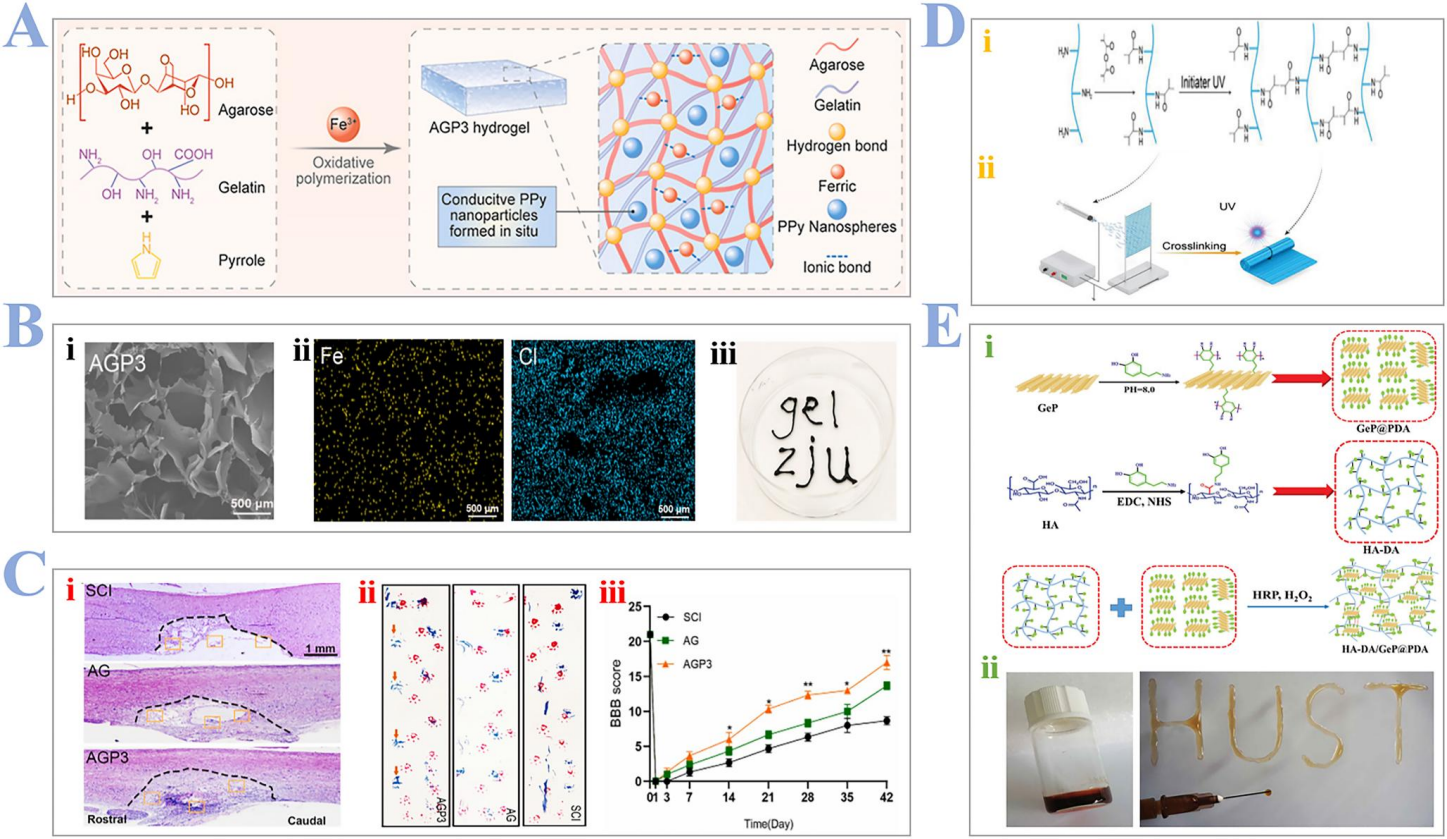
(A) Schematic illustration of preparation of the AGP3 hydrogels for regeneration of SCI. (B) (i) SEM images of high pyrrole content (AGP3) in hydrogels. (ii) Element analysis of AGP3 hydrogels via Energy dispersive X‐ray (EDX). (iii) Injection of AGP3 hydrogels by a 26‐G syringe. (C) In vivo functional recovery evaluation. (i) Representative H&E staining images of the spinal cord in each group. (ii) Images of hind limb (blue) and forelimb (red) footprints in different groups. (iii) BBB score of different groups at different time. Reproduced with permission.^78^ Copyright 2022, The Authors, published by Elsevier. (D) Schematic illustrations of the preparation process of GelMA hydrogel scaffold. (i) Representing the chemical modification and crosslinking process. (ii) Demonstrating the electrospinning process. Reproduced with permission.^79^ Copyright 2019, Wiley. (E) (i) Schematic illustrations of the preparation process of GeP@PDA sheets, HA‐DA polymers, and HA‐DA/GeP@PDA hydrogels. (ii) The image of HA‐DA/GeP@PDA hydrogels injected using a 26‐G syringe. Reproduced with permission.^80^ Copyright 2021, Wiley. BBB, blood–brain barrier.

Biao Yang and coworkers^78^ recently proved a conductive (FeCl_3_) supramolecular hydrogel could effectively facilitate the SCI regeneration. They prepared the AGP3 hydrogels by mixing FeCl_3_, agarose, gelatin, and pyrrole together to achieve similar modulus and conductivity as that of the spinal cord, as shown in Figure 5A. The concentrations of pyrrole are designed to regulate the mechanical properties of the hydrogel. Furthermore, the introduction of Fe^3+^ can increase the crosslinking densities of hydrogels, as shown in Figure 5B. They further used H&E staining and behavioral assessment to prove both qualitatively and quantitatively that the synthesized hydrogel can promote the functional regeneration of the spinal cord in vivo, as shown in Figure 5C.

Chunmao Chen and coworkers^79^ constructed an electro‐spun hydrogel fiber by using another approach different from Biao Yang et al. They chose UV to enable crosslinking between GelMA and gelatin, as shown in Figure 5D. They demonstrated that hydrogel fibers can facilitate the proliferation, differentiation, and migration of endogenous cells as well as directional extension of axons. Chao Xu and colleagues^80^ suggested phosphide‐reinforced hydrogel scaffolds that can enhance SCI regeneration by activating endogenous NSCs to enter into the neurogenesis phase at the lesion site and inducing immune regulation. They fabricated this scaffold by selecting germanium phosphide/polydopamine (GeP@PDA) as nanosheets and hyaluronic acid (HA) as the scaffold matrix, as shown in Figure 5E. The advances of biomaterials with adaptable mechanical and chemical properties can help enhance cell survival and guide tissue regeneration in vivo. Their sensitive response to surrounding environment (temperature, light, stress, etc.) can simulate a more authentic scenario in real life.

However, it is worth noting that although hydrogels have many outstanding properties as discussed above, several problems remain to be solved. For example, the poor biodegradation characteristic results in a long preservation time, which hinders the regeneration of neurons and the reconstruction of ECM. What is more, several improvements could be made. The controlled release of therapeutic drugs combined with hydrogels have already been illustrated to have optimal clinical benefit.^81^ As time goes on, the real‐time state of SCI is different from that when hydrogels are injected, thus it is ideal if we could fabricate hydrogels that can release different amounts and types of drugs according to the stage of SCI recovery.^76^

### 5.2 Scaffold made by 3D bioprinting technique

Three‐dimensional (3D) printing also referred as additive manufacturing is currently a thriving research direction in tissue engineering. Recently, breakthroughs have been made to enable 3D printing of biomaterials, living cells, and biochemicals. In comparison with printings in other areas like art, engineering, and bioprinting involves more complexities, because factors like biocompatibility (toxicity) of materials and limitations of technologies are closely related to the survival and vitality of cells as well as the successful reconstruction of tissues.^82^ Although advances have been made by the combination of techniques from physics, cell biology, chemistry, certain issues like drug delivery, toxicology, resolution, high throughput still remain unsolved.^83^ Apart from challenges of shifting and adapting techniques designed in other areas to printing fragile, living bio‐materials, the largest challenge is the construction of scaffolds that resemble ECM with comparable functions. Here, we explore different areas of 3D bio‐printing by introducing different types of bio‐scaffolds and recent advances.

#### 5.2.1 Types of 3D scaffolds based on biomaterial

In the beginning, 3D printing techniques were mainly used in nonbiological areas like architecture, art, engineering, etc. Consequently, metals and synthetic plastics are the main materials, but for printing bio‐scaffolds, these materials are incompatible and toxic to living cells. Fortunately, in recent years,^84^ materials with good biocompatibility as well as mild printing methods that do minimum harm to living cells and tissues have been found.

In the area of regenerative medicine, materials used for repair are mainly divided into natural polymers and synthetic molecules. HA, alginate, collagen, gelatin, preprocessed animal tissues, etc., belong to the natural group.^85^ They resemble the biological characteristics of ECM. PLGA, PEG, Poly(D,L‐lactide‐co‐ glycolide) (PCL), etc., belong to the synthetic group.^86^ Although they may have poor biocompatibility, they have adequate mechanical properties and can be tailored according to patients' specific needs. Considering the complexity of human organs and tissues, bio‐printed tissues should maintain their mechanical properties and geometrical shape in the long term, avoiding collapse in structures. What is more, bio‐printed tissues should have a sensitive response to the surrounding environment in vivo, changing its physical and biological properties to facilitate functional regeneration. In conclusion, materials used in bio‐scaffolds must have the following traits^82^: printability, biocompatibility, dynamic mechanical properties, biomimicry, controllable degradation kinetics, etc.

#### 5.2.2 Synthetic scaffolds

**FIGURE 6:**
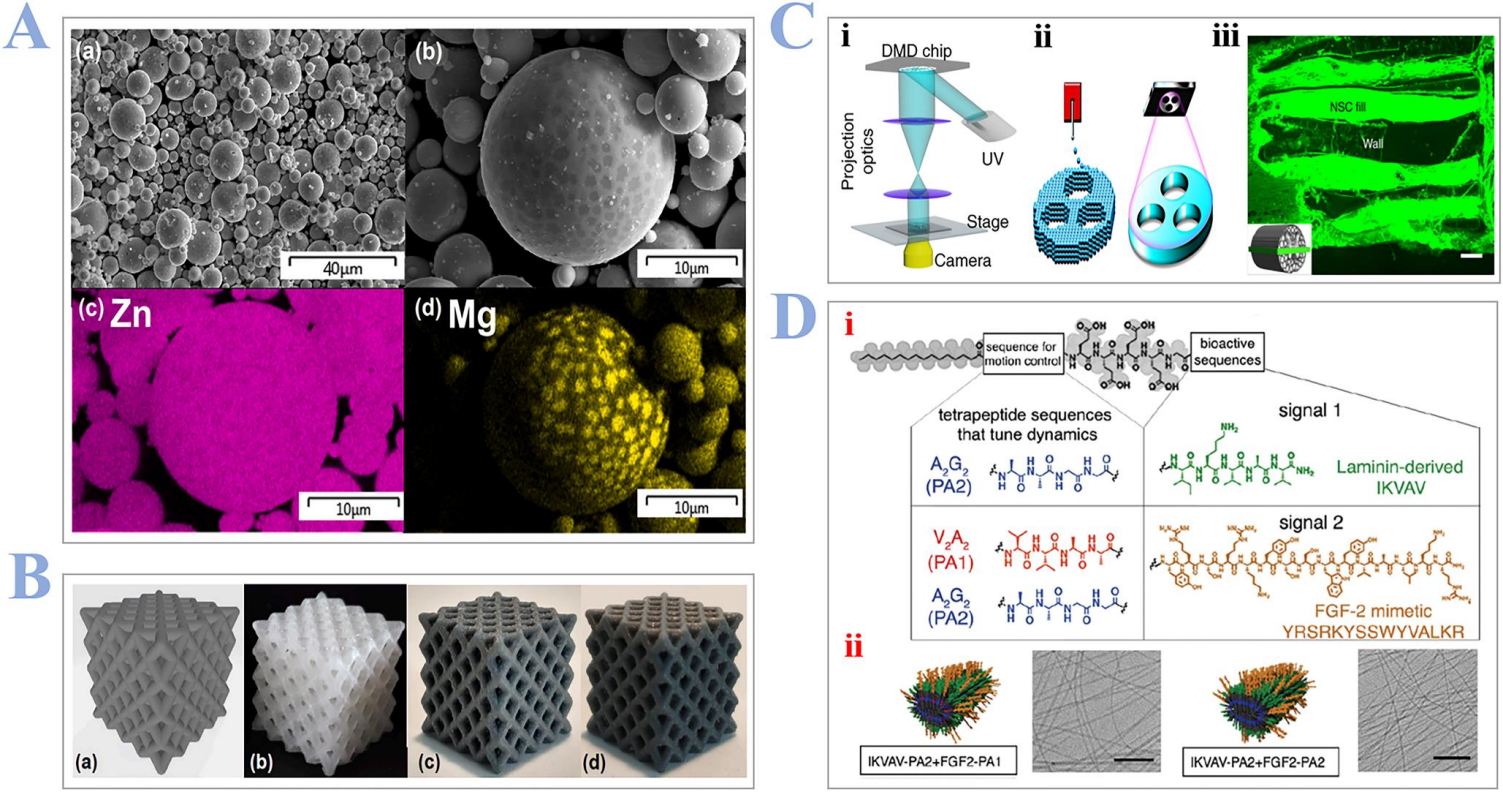
(A) (a,b) SEM images, Zn and Mg powder. (c,d) Energy dispersive spectrometer analysis demonstrating the allocation of Zn/Mg. (B) Scaffolds made from PLA/Zn, fabricated by FFF. (a) CAD model, (b) Scaffolds made from PLA, (c) PLA and addition of 3.5% Zn and (d) PLA and addition of 10.5% Zn. Reproduced under terms of the CC‐BY license.^88^ Copyright 2022, The Authors, published by Elsevier. (C) (i) setup of the 3D printer using microscale continuous projection printing (μCPP) methods. (ii) μCPP layer‐less 3D printing methods can create structures with continuous layers compared with extrusion‐based method. (iii) Channels are filled with NPCs (GFP positive). Reproduced with permission.^72^ Copyright 2019, The Authors, published by Springer Nature. (D) PA scaffolds with two identical sequences, different in the chemical structure. (i) Chemical structures of the different PA molecules applied. (ii) Molecular illustrations of nanofibers demonstrating two bio‐signals. TEM pictures, IKVAV PA2 co‐assembled with FGF2@PA1 and FGF2@PA2. Reproduced with permission.^89^ Copyright 2021, AAAS. 3D, three‐dimensional; CAD, computer‐aided design; NPC, neural progenitor cells.

As discussed above, scaffolds made by synthetic materials can have controllable mechanical properties and geometrical shape, making them widely used in regenerative medicine. Widely used materials^87^ are metallic biomaterials (CoCr, Ti‐6Al‐4V, etc.), polymeric biomaterials (PCL‐PLGA, PCL, etc.), and ceramic biomaterials (HA, bio‐glass, etc.). Overall, synthetic scaffolds can be further divided into two groups: nonbiodegradable and biodegradable. Recent studies have focused on the application of biodegradable scaffolds as it can provide a more biologically friendly environment than nonbiodegradable ones. Pascual‐González and colleagues^88^ developed a novel poly‐lactic acid/Zn bio‐scaffold with ideal biodegradable properties. They also confirmed that the scaffolds are with similar stiffness, strength, and stressing ability as PLA scaffolds which have the feasibility to be reinforced with Zn particles. PLA filaments are strengthened with a different weight gradient of micrometer‐sized Zn/Mg alloy particles, as shown in Figure 6A. Different concentration rations of scaffolds are shown in Figure 6B. Jacob Koffler and colleagues^72^ reported a novel 3D printing method (microscale continuous projection printing) to print scaffolds that are scalable to the spinal cord, as shown in Figure 6C(i,ii). They further encapsulate NPC in the printing materials (Figure 6C(iii)) and confirmed the successful restoration of motor function. Distinctively, Álvarez et al. fabricated a peptide fibril scaffold which bears dual‐peptide sequences promoting neural regeneration, as shown in Figure 6D(i). One sequence can reduce glial scarring and another sequence can promote the formation of blood vessels. The synthesized scaffold has two unique bio‐signals: the laminin signal IKVAV, known to promote differentiation of NSCs into neurons and to extend axons and the fibroblast growth factor (FGF) peptide YRSRKYSSWYVALKR, known to activate the FGFR1 receptor and facilitate the proliferation of NSCs, as shown in Figure 6D(ii). The two signals (FGF‐2 and IKVAV) were placed at the termini of two different peptides with alkyl tails, known as peptide amphiphiles (PAs), that copolymerize noncovalently in aqueous media to form supramolecular fibrils.

#### 5.2.3 Natural tissue scaffolds

**FIGURE 7:**
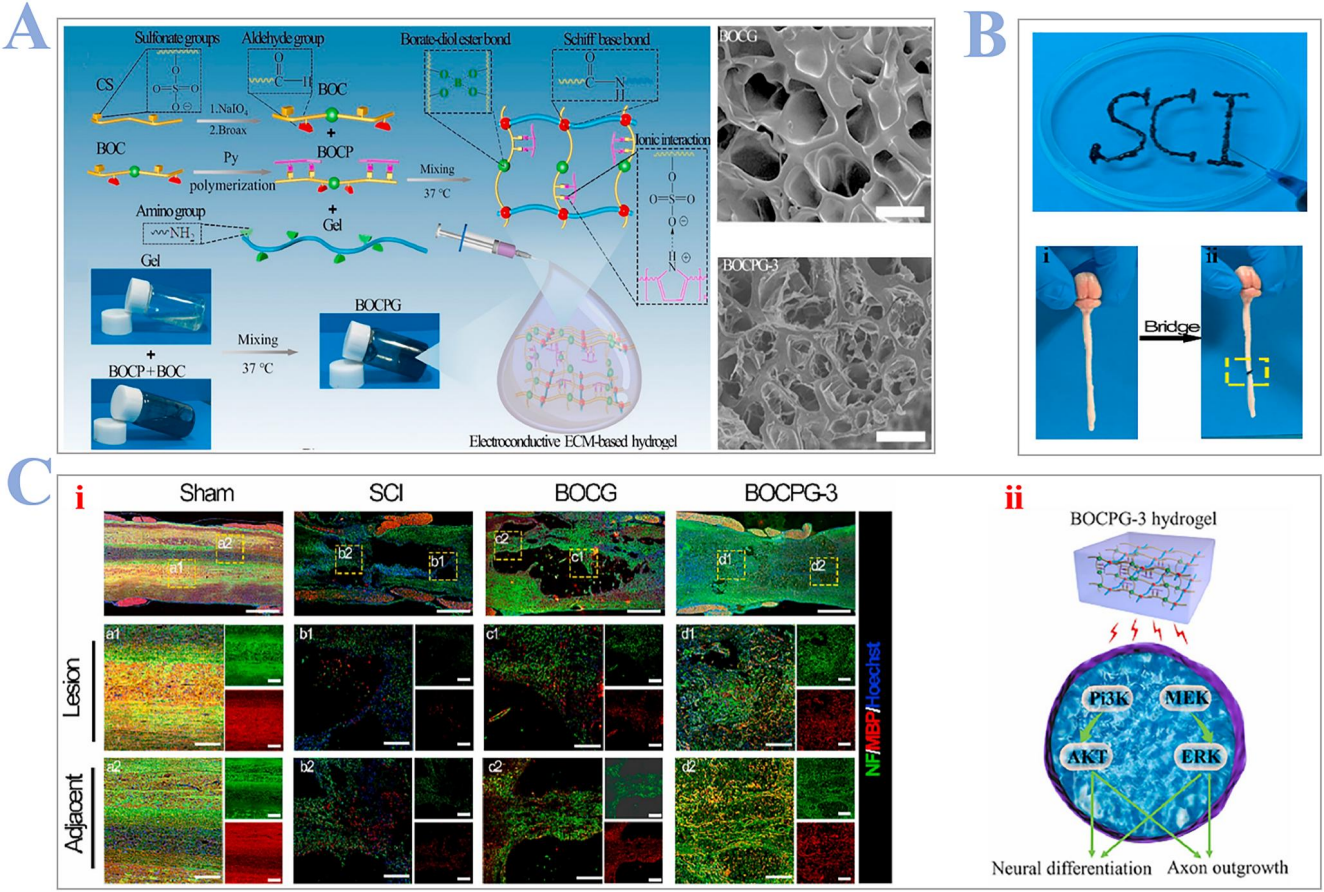
(A) Schematic illustrations of the formation of BOC, BOCP, and BOCPG molecule; SEM images, BOCPG‐3 and BOCG. Scale bar: 25 μm. (B) Injection of BOCPG‐3 hydrogel by a syringe. (i) Spinal cord of mouse, (ii) hydrogels were used to fill the injured area in the lesion area, showing the adhesive feature of hydrogels. (C) Injection of BOCPG‐3 hydrogels promote the regeneration of axon. (i) Immunofluorescence images, stained with antibodies, green (neurofilament) and red (myelin protein). Scale bar: 1 mm. (ii) Illustration demonstrated the process of BOCPG‐3 hydrogel promoting the outgrowth of axon through PI3K/MEK, etc., pathway. Reproduced with permission.^93^ Copyright 2022, The Authors, published by Elsevier. BOC, borax oxidized chondroitin sulfate; BOCP, BOC‐doped poly‐pyrrole; BOCPG, BOC‐doped poly‐pyrrole dotted with gelatin.

Natural tissues are composed of living cells, biomolecules, and ECM.^9^ ^a^ The preparation process of natural tissue scaffolds is simple. Among them, ECM is one of the most widely used 3D bio‐printing materials, because ECM or dECM mimics are considered to simulate a more similar 3D environment for surrounding cells in mechanical, biological, and geometrical level. ECM is a hydrophilic and anisotropic 3D matrix that consists of HA, chondroitin sulfate (CS), proteins (collagen, elastin, and fibrin), etc.^90^ There are two major types of ECM: ECM mimics (Silk fibroin, chitosan, self‐assembling peptides, etc.) and processed ECM (Gelatin, decellularized ECM, etc.). Among them, decellularized ECM (dECM) is one of the most effectively bioink sources because of its ideal properties.^91^ dECM are made from natural ECM where the native cells are removed by chemical, physical, and enzymatic methods in order to avoid immune response in the host body. However, the dECM retains its hydrophilic, anisotropic, and biomechanical properties. The processed ECM serve as a cell friendly micro‐environment that provide conducive cues like growth factors, differentiation factors, etc., to the host cells.^92^ An interesting biomaterial using ECM biopolymer for SCI repair is fabricated by Luo and coworkers.^93^ They developed a novel injectable, electroconductive hydrogel with self‐healing functions. The hydrogel is made of a natural ECM biopolymer (chondroitin sulfate and gelatin) containing poly‐pyrrole, while vesting it with electroconductive properties. They homogeneously mixed the borax oxidized chondroitin sulfate (BOC), BOC‐doped poly‐pyrrole (BOCP), and gelatin together to form the BOC‐doped poly‐pyrrole dotted with gelatin (BOCPG) and ECM‐based hydrogel (Figure 7A). They also demonstrated the injection of this material in vitro (Figure 7B) before conducting in vivo experiments. They further confirmed that the ECM‐based hydrogel realizes its function by promoting axon outgrowth and neural differentiation, as shown in Figure 7C. In conclusion, ECM‐based hydrogels can have a high water content and highly porous structure, which can facilitate the local ECM remodeling. What is more, due to the fact that signal transmission between cells occurs mainly through the ECM, ECM‐based hydrogels can thus help enhance endogenous NSCs migration, neuronal differentiation, and myelinated axon regeneration.

In conclusion, although ECM‐based bioinks have various benefits, individual ECM components alone cannot satisfy our requirements of what is needed for SCI regeneration. As Van Day Truex once stated: “In design, Mother Nature is our best teacher.” We should draw inspirations from nature to guide the regeneration strategies for tissues and organs.

### 5.3 Graphene‐based materials

The hexagonal honeycomb lattice of graphene, a two‐dimensional carbon nanomaterial, is made up of carbon atoms with sp^2^‐hybridized orbitals. Great strength, high flexibility, and bendability, super hydrophobicity, super lipophilicity, and strong thermal conductivity are all qualities of graphene.^94^ Due to its unique combination of features, graphene is now an excellent choice for interfaces that aim to record, modify, and regenerate delicate neural tissues, such as the spinal cord. In fact, raw graphene is being used in potential advanced therapies for the treatment of SCI, which continues to be one of the most disruptive medical disorders, along with other graphene‐based materials (GBMs) such as graphene oxide (GO) and reduced graphene oxide (rGO). GBMs have shown promising outcomes in the following areas^95^: (1) the capacity of GBMs to support proper axonal sprouting and long‐tract outgrowth as well as to enhance NSC survival and differentiation; (2) the capacity of 3D graphene‐based scaffolds to alter the spinal cord's hierarchical structure and encourage a successful bridging of the lesion site; and (3) their usage in nerve tissue engineering scaffolds as seed cell, trophic factor, and medication transporters to establish a foundation for creating a local microenvironment following spinal cord damage.

**FIGURE 8:**
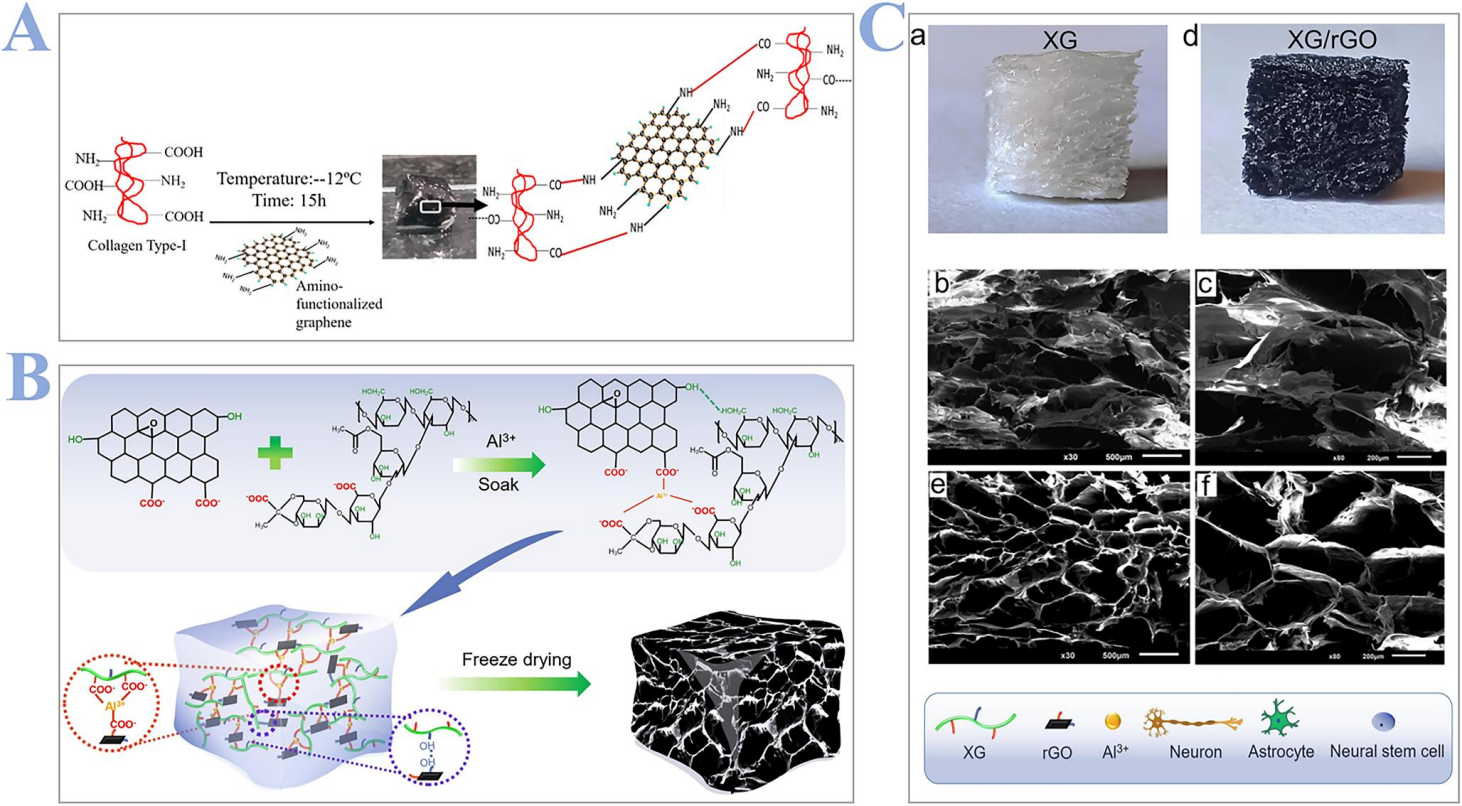
(A) Schematic illustration of the preparing process of graphene‐crosslinked collagen cryogel. Reproduced with permission.^96^ Copyright 2021, Elsevier. (B) Synthesis mechanism and process of the XG/rGO gel scaffold. (C) The porous structure of scaffolds (a) appearance of XG scaffold; (b,c) SEM and high magnification SEM image of XG scaffold; (d) appearance of XG/rGo scaffold; (e,f) SEM and high magnification SEM image of XG/rGo scaffold. Reproduced with permission.^98^ Copyright 2022, Elsevier. rGO, reduced graphene oxide; XG, xanthan gum.

Recently, many studies have focused on this unique material. For example, Agarwal and coworkers^96^ fabricated graphene‐crosslinked collagen cryogels with high electroconductivity and ideal immunomodulatory properties for SCI regeneration. They first prepared the collagen type‐I and then formed the super macroporous cryogel by carbodiimide chemistry at sub‐zero temperature where the crosslinking between the ‐COOH of collagen and ‐NH2 of amino‐functionalized graphene occurred (Figure 8A(i)). Similarly, in another study by Agarwal in 2022,^97^ they further validated the potential of fabricated graphene collagen (Gr‐Col) cryogels in polarizing astrocytes, microglia, and fostering neuronal regeneration. Interestingly, different from Agarwal, Xue and coworkers^98^ used xanthan gum (XG) to modify graphene (Figure 8B). They fabricated a reduced graphene oxide composite XG via the freeze‐drying technology. They first mixed the solution of XG and rGO together. Afterward, AlCl_3_ solution was uniformly spread in the gel mold before freeze‐drying to obtain the porous structure (Figure 8C). They confirmed that scaffolds made by such an approach could promote nerve regeneration, inhibit glial scar formation, and facilitate locomotor recovery in rats.

Although GBMs have good biosafety and biocompatibility, as well as outstanding effectiveness of improving electrical conductivity and supporting axonal regeneration, several changes are still faced by GBMs. Given the lack of ongoing clinical trials examining the effectiveness and biosafety of GBMs in patients with SCI, as well as the typical length of time and expense required to have a drug or medical device approved by the government, it is evident that regenerating the injured spinal cord using graphene‐based platforms still has a long way to go. Furthermore, despite being biosafe in experiments, some scientists point out it is still unclear if graphene and products based on it are biosafe in human use, in which case a standard for safe dosage of GBMs used in clinical trials is needed. These safety issues limit the use of such materials in the therapeutic management of SCI.

## 6 BIOMOLECULE‐BASED STRATEGIES FOR SCI REGENERATION

Complex molecular and cellular mechanisms are needed to repair the injury sites of the spinal cord. Unfortunately, amounts of endogenous biomolecules are relatively too low to exert a positive effect on the regeneration of neurons at the injury sites. However, the introduction of exogenous biomolecules is a versatile and novel approach to promoting functional recovery of the spinal cord.^99^ Biomolecules can activate a series of downstream signaling cascades by binding with corresponding receptors. What is more, with the help of various scaffolds mentioned above, the disadvantages of simple biomolecules like short half‐life, fast degradation rate, etc., can be solved. The scaffolds with good biocompatibility serve as a suitable vehicle for these biomolecules to support SCI regeneration by providing a steady release environment for them.^100^

In conclusion, although the remarkable breakthrough of tissue engineering has proved to play a crucial part in the regeneration of SCI. Many of these scaffolds cannot demonstrate their real potential when applied in vivo.^101^ To optimize scaffolds' properties, recent research efforts^102^ have been concentrated on the introduction of biomolecules to facilitate the scaffolds' function. Additionally, scaffolds in turn can also facilitate the biomolecules' function. The following sections will focus on two groups of biomolecules (growth factors and exosomes) and the drug delivery system of CNS.

### 6.1 Growth factors

Growth factors (GFs) are polypeptides that can facilitate proliferation, differentiation, and migration of living cells in the CNS.^103^ GFs exert their functions mainly by triggering downstream signaling pathways through receptors. The most common GF family members are BDNF, FGF, nerve growth factor (NGF), insulin growth factor (IGF), etc.^104^ For example, several studies have illustrated that NGF‐loaded nerve guidance conduits (NGCs) could promote the survival of neurons and outgrowth of axons, showing the positive effect for both central nerve and peripheral nerve repair.^105^

**FIGURE 9:**
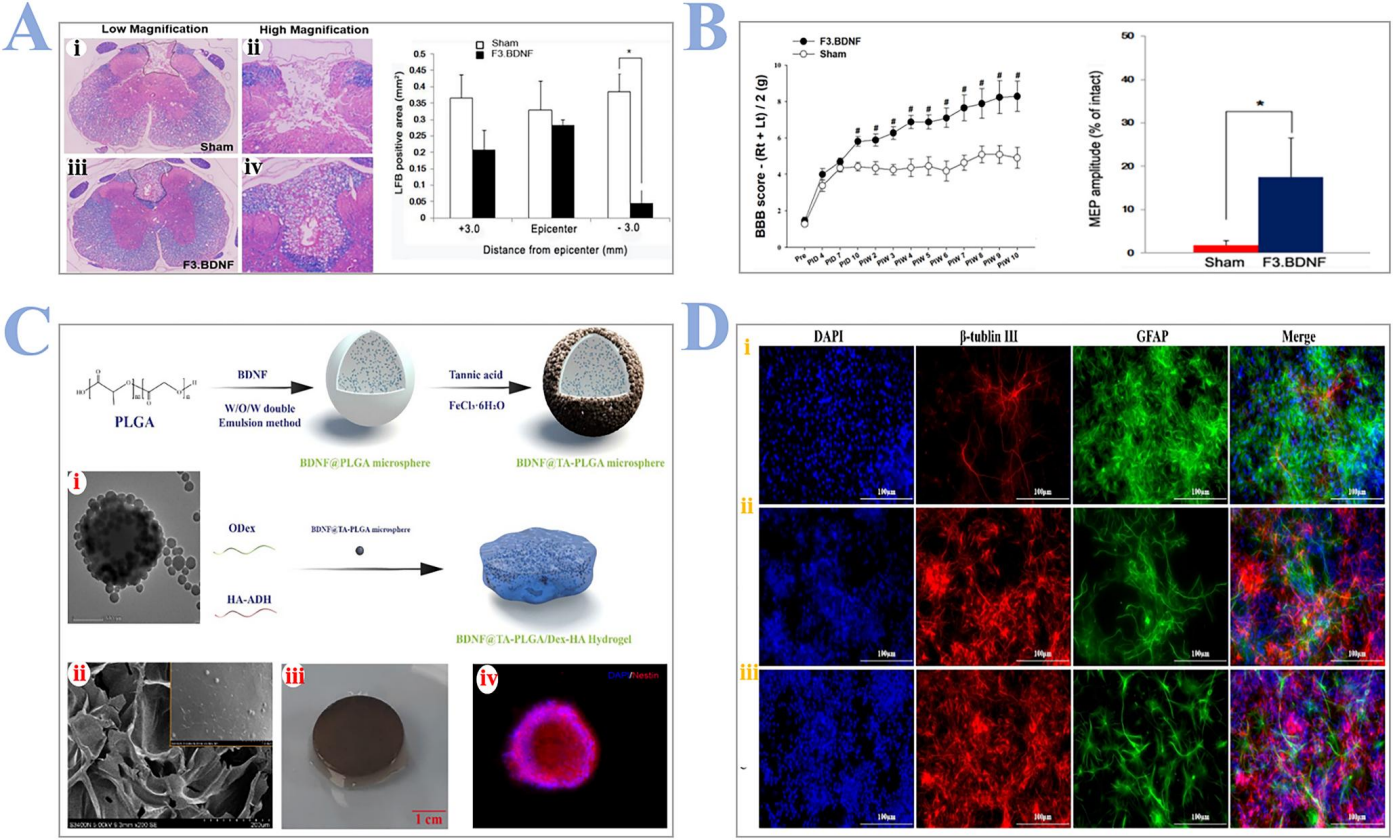
(A) Changes of the dorsal column in the experimental group, compared with the sham group. (i–iv) Representative luxol fast blue (LFB) staining images (low and high magnification) of the injured spinal cord (traverse section) and quantitative analysis of the LFB‐positive area. (B) Motor function assessment in the hindlimbs and values of average amplitudes of motor‐evoked potential. Reproduced under terms of the CC‐BY license.^108^ Copyright 2021, The Authors, published by Multidisciplinary Digital Publishing Institute. (C) Schematic illustrations of the preparation process of BDNF@TA‐PLGA/Dex‐HA hydrogels. (i) TEM images, BDNF@TA‐PLGA microspheres. (ii) SEM images, BDNF@TA‐PLGA/Dex‐HA hydrogel. (iii) Digital photos, BDNF@TA‐PLGA/Dex‐HA hydrogel. (iv) Immunostaining images, NSCs with nestin (red) and DAPI (blue), magnification: ×400. (D) Images of NSCs differentiation in each group after 7 days of incubation: (i) Dex‐HA hydrogels, (ii) BDNF@PLGA/Dex‐HA hydrogels, and (iii) BDNF@TA‐PLGA/Dex‐HA hydrogels into Tuj1‐positive cells, for neurons (red) and GFAP‐positive cells for astrocyte (green). Cell nuclei, DAPI (blue). Reproduced with permission.^109^ Copyright 2021, Elsevier. BDNF, brain‐derived neurotrophic factor; NSC, neural stem cells.

BDNF, as one of the most studied neurotrophic factors in human, signals through tyrosine kinase receptor B(TrkB). It helps phosphorylate TrkB and activates the downstream signaling cascades, reducing the apoptosis of neurons in the spinal cord.^106^ It is considered to serve as a functional mediator to restore structural cues of CNS. It can also protect the injured spinal cord from neurodegeneration and promote proliferation and regeneration of various neurons (sensory neurons, dopaminergic neurons, etc.) at injury sites.^107^ Da‐Jeong Chang and colleagues^108^ developed genetically modified hNSCs with overexpressed BDNF to confirm their role in the functional regeneration of SCI. They found that overexpressed BDNF can help decrease the demyelinated dorsal column significantly (Figure 9A), and rats with BDNF displayed better motor function and electrophysiological recovery (Figure 9B). Fei Huang and colleagues^109^ proved that the introduction of BDNF in vitro can promote NSCs differentiation and inhibit astrocyte differentiation. They encapsulated BDNF in a carrier made of PLGA, forming a tannic acid (TA) modified microsphere, then hydrogels fabricated with oxidized dextran (Dex) and HA‐hydrazide (HA‐ADH) was mixed with the previous microspheres, as shown in Figure 9C. They confirmed the effectiveness of BDNF@TA‐PLGA/Dex‐HA hydrogel by comparing confocal microscopy images of different experimental groups, as shown in Figure 9D.

FGF can facilitate the regeneration and survival of various CNS and PNS neurons both in vivo and in vitro.^110^ FGFs can be divided into two groups: acidic FGF (aFGF) and basic FGF (bFGF) according to its isoelectric characteristics. bFGF is more widely used as most cells cannot synthesize and secrete aFGF. bFGF can support the growth of neurons and the extension of axonal. bFGF possibly exerts its function by slowing down the apoptosis of neurons and by stabilizing the ion levels (calcium, ferric, etc.) at the injury sites.^111^ They may also reduce the formation of glial scar as reported by Cao et al.^112^ Karina P. Reis and colleagues^113^ confirmed that FGF can facilitate the survival of neurons and promote the stimulation of neurogenesis by constructing FGF‐encapsulated PLGA microfibers.

IGF is also an important GF, which can facilitate the regeneration and proliferation of NSCs by slowing down the apoptosis of nerve cells and directing the migration of NSCs. Su Pan and colleagues^114^ combined FGF and BDNF together and proved that this combination coated with PLGA/GO material can protect NSCs from oxidation, enhance proliferation and differentiation of NSC in vitro. Additionally, this novel combination can facilitate functional locomotor recovery and reduce the formation of cavities.

### 6.2 Exosomes

As mentioned in the “Cell‐based strategies for SCI recovery” section, although MSC can reduce injured area and promote extension of axonal, its application is limited by heterogeneity, immunogenicity, and tumorigenicity. Recent research,^115^ however, show that exosomes can have huge potential in the functional regeneration of SCI. Recently, a type of intercellular communication has drawn much attention.^116^ Cells use extracellular vesicles (EV) to exchange information in this communication pathway. Exosomes as well as the apoptotic body and microvesicle are three main types of EVs.^117^ EVs contain useful molecules like DNA, miRNA, transcription factors, etc. Consequently, EVs play an important role^118^ not only in intercellular communication but also in treating many diseases like SCI.

**FIGURE 10:**
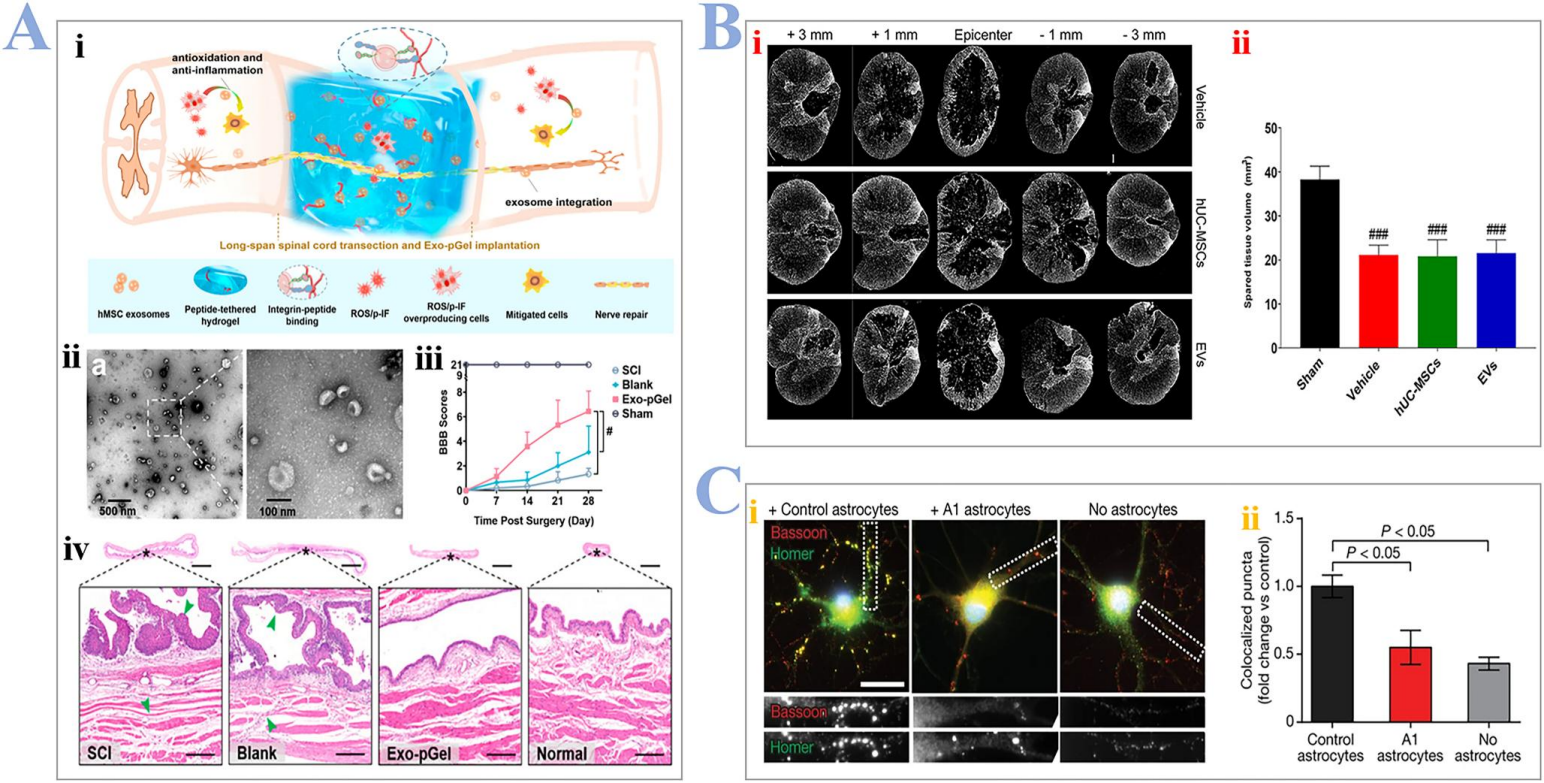
(A) (i) Schematic illustrations of Exo‐pGel for SCI treatments. (ii) TEM images of morphology of human mesenchymal stem cell (hMSC)‐derived exosomes. (iii) BBB scores of each group of rats during 28 days post‐surgery. (iv) HE staining of bladder tissues. Reproduced with permission.^123^ Copyright 2020, ACS Publications. (B) (i) Representative images of GFAP along the rostro–caudal axis (cross section) of the spinal cord. Scale bar: 200 µm. (ii) Total volume of spared spinal cord parenchyma. Reproduced under terms of the CC‐BY license.^120^ Copyright 2019, The Authors, published by Frontiers Media S. A. (C) (i) Retinal ganglion cells (RGCs) with/without A1 astrocyte, immune‐stained with postsynaptic and presynaptic markers bassoon (red), homer (green). Colocalization was considered as a structural synapse (yellow). (ii) The group with A1 astrocytes demonstrated 50% less synapses, compared with groups with resting astrocytes. Reproduced with permission.^121^ Copyright 2017, Springer Nature. BBB, blood–brain barrier; SCI, spinal cord injury.

MSCs‐derived exosomes are almost free of heterogeneity, immunogenicity, and tumorigenicity and are with little ethical and moral issues compared with only MSCs. What is more, thanks to the small volume of exosomes, they can easily penetrate through the BSCB without being captured by surrounding tissues (liver and lung tissues, etc.).^119^ These properties combined make MSCs‐derived exosomes more suitable for SCI repair. In this circumstance, exosomes undisputedly attract much more attention than MSCs. The derived exosomes can regulate the relative concentration of anti‐inflammatory and inflammatory factors (interleukin‐1β, etc.), and the tumor necrosis factor (TNF‐α).^115^ ^b^ In this way, it can have anti‐inflammatory effects at the injured sites. Li et al. constructed an adhesive hydrogel modified with peptide (Exo‐pGel), which can also immobilize the human MSC‐derived exosomes, as shown in Figure 10A(i,ii). The encapsulated exosomes demonstrate effective and sustained release in the injury sites, promoting overall motor recovery and tissue regeneration, as shown in Figure 10A(iii,iv). In another interesting study, Romanelli and colleagues reported the injection of umbilical cord mesenchymal stem cells (UCMSC‐exosomes) that can quench the inflammatory response, interact directly with rat microglia, and reduce scarring response. The volume of the remaining intact spinal cord was also studied, and groups treated with UCMSC‐exosomes had lower spared tissue volume compared with the sham group, as shown in Figure 10B. The exosomes can also promote the polarization of macrophage.^120^ In other words, they can reduce the emergence of M1 macrophages (promote tissue inflammation) and increase M2 macrophages (reduce tissue inflammation). Similarly, exosomes can reduce A1 astrocytes and increase A2 astrocytes. A1 astrocytes are toxic to myelin, neurons, etc. On the other hand, A2 astrocytes can upgrade the secretion of neurotrophic factors. Interestingly, Liddelow and colleagues^121^ confirmed the secretion of Il‐1α, TNF facilitate the emergence of A1 astrocytes which can secrete a neurotoxin, accelerating the death of surrounding cells. Also, A1 astrocytes do not preserve the normal astrocytic functions, as shown in Figure 10C. But why the injured CNS would facilitate the production of a kind of neurotoxic astrocyte remains unknown. Finally, the MSC‐derived exosomes can protect BSCB from SCI. As the almost inevitable result of SCI, the blood vessels near the injury sites are destroyed immediately, afterward the integrity of BSCB is further damaged. Matsushita and colleagues^122^ demonstrated that the intervention of MSCs help restore the integrity of BSCB by restoring the vascular permeability to its original level.

### 6.3 Importance of drug delivery in SCI recovery

When it comes to the regeneration of the injury sites in SCI, the blood–brain barrier (BBB) is a problematic barrier to overcome and is the key to the effectiveness of above strategies.^124^ There are many factors influencing the effectiveness of drug delivery across BBB. Therefore, the achievement of sufficient drug delivery across BBB is crucial in treatments for CNS disorders like regeneration of the spinal cord. Although drugs like biomolecules (GFs, exosomes, etc.) and cells can have various functions and facilitate the regeneration of the spinal cord in many ways as mentioned in the above sections, most of these functions are applicable only in vivo.^9^ ^b^ In order to fully tap their potential, the efficient delivery system for drugs and cells is necessary. Recently, increasing research efforts have focused on the delivering therapeutics of drugs to the CNS.^125^ Brain delivery techniques are grouped into two categories: invasive and non‐invasive techniques.^124^ Brain stimulation, brain injection, and intracerebral grafts are invasive ones. Focused ultrasound, nanoparticulate system, and biological‐based systems are noninvasive ones. We focus on the latter in this section.

Neurotropic viruses have a special tendency to attack nerve cells, they have evolved various methods to lurk into the brain such as hiding inside the immune cells, etc.^126^ Therefore, scientists can use gene editing techniques to deliver drugs across BBB through neurotropic viruses. However, nanoparticulate systems are the most widely used approach to delivering drugs.^127^ Liposomes are known for their properties: reduced toxicity and increased accumulation at the target sites.^128^ Wang and colleagues^129^ developed a scar‐homing liposome for the functional regeneration of SCI. The liposomes prolong the half‐life of the encapsulated drugs and initiate the myelination of injured axons. Other drug delivery systems like polymeric nanoparticles have also shown success in preclinical trials.^130^

## 7 OTHER STRATEGIES FOR SCI RECOVERY

### 7.1 Acupuncture treatment combined with rehabilitation training

Acupuncture is a unique Chinese method of treating diseases. The core principles of this technique are to use external interventions to treat internal diseases, through the conduction of meridians and acupoints, and the use of certain operation methods, to treat systemic diseases. Thanks to its unique advantages: easy operating method, little side effects, low hospitalization costs, etc., in the Tang dynasty, Chinese acupuncture had spread to Japan, India, and other countries. Acupuncture can be divided into moxibustion, cupping, and modern acupuncture (microwave stitch, acupoint ion penetration, etc.). Rehabilitation training is the key to clinical treatment for SCI and has been proved to facilitate functional recovery in patients with SCI.^131^ Rehabilitation training can upregulate GFs (BDNF, etc.) level, enhance neural connections, and facilitate axonal sprouting.^132^ Xiong and colleagues^133^ combined acupuncture therapy together with rehabilitation training and conducted trials with three groups of patients (24 patients each). The results showed that acupuncture increased Modified Barthel Index and ASIA Motor Score, but it seemingly has no significant effects in improving the sensory performance in patients with SCI. Additionally, the treatment frequency of 5 per week is safer and more effective than that of 3 per week. However, it should be noted that there might exist a placebo effect on the study groups as all the participants were Chinese people. Recent studies have also integrated robotic technology with the regenerative medicine field. Tamburella and colleagues^134^ used Overground powered lower limb exoskeletons (EXOs) for the post‐surgery recovery of patients with SCI. However, more studies need to be carried out to establish clear evidence about all possible EXOs' advantages and disadvantages.

### 7.2 Combinational strategies

Many factors as mentioned above, especially the formation of glial scar, insufficient supplement of GFs, extensive death of cells, etc.,^20^ make the functional regeneration of the spinal cord still a tricky problem regardless of various advanced treatments. Therefore, to tackle these complicated syndromes, combining cell‐based, biomolecules‐based, and biomaterials together is of same importance as developing novel strategies. For example, Fan and colleagues developed an electroconductive hydrogel loaded with exosomes, they found that this material can regulate the immune system and enhance growth of myelinated axon more effectively than exosomes or hydrogel alone. Li and colleagues^135^ used MnO_2_ nanoparticles to coat hydrogel and confirmed that the encapsulation of MnO_2_ can alleviate the oxidative environment better than hydrogel alone. Other combinations, like using cells and scaffolds along with pharmacological agents or other biomolecules (GFs, exosomes, etc.) have already been covered in recent advances in the above sections.

## 8 CONCLUSION AND PERSPECTIVE

We have by far summarized the advances of three main strategies with multiple combinations for drug delivery and regeneration of the spinal cord. Cell‐based strategies were presented with their outstanding neuroprotective and neuroregenerative characteristics introduced in length, following the review of scaffold‐based strategies represented by hydrogel and ECM‐derived scaffolds for their application in SCI repair. Finally, biomolecule‐based strategies were presented and vehicles loaded with FGF, exosomes, etc., were comprehensively discussed. However, regardless of remarkable advances made in recent years in the area of SCI repair with various novel strategies, the successful functional regeneration of the injured spinal cord remains a complicated physiological process. Several critical issues remain to be solved for successful clinical translations from animal models to human.

The first problem is concerning the construction of ideal biomaterials for either the encapsulation of stem cells, construction of scaffolds, or drug delivery vehicles. Especially in recent years, drawing inspiration from nature, scientists have explored various man‐made scaffolds for SCI repair by combining natural‐derived materials. However, the natural component for tissue regeneration in these biomaterials have several lethal drawbacks. For example, natural materials typically have poor mechanical properties and a fast degradation rate in vivo which make them unable to endure drastic force in activities such as running. One possible solution is to continue exploring biomaterials with desirable characteristics. For example, it is of crucial importance to construct scaffolds with controllable drug release rate. What is more, the degradation rate of the scaffold should be synchronous with the regeneration rate of the spinal cord in order to minimize secondary injury in the transplantation of scaffolds.

Second, although various studies have been conducted in recent years, scientists still have not come up with a clinical effective strategy for the treatment of SCI. The complex syndromes of SCI and the plain fact that most studies are decentralized are accounted for in this phenomenon. On one hand, in many studies the variations are based on an empirical way. For example, there is still no precise principle to decide the injected cell numbers, cell types, etc., which means there exists no universal standard for cell‐based therapy in clinical trials. On the other hand, although the use of scaffolds along with cells can improve the effectiveness of SCI therapies significantly, biomaterials still face many challenges. For example, the preparation process is still too complex and time‐consuming for many biomaterials. Mass and stable production of biomaterials have not been realized yet. What is more, even though these strategies alone demonstrate conducive effects on the regeneration of the spinal cord, they fail to have their full potential displayed when applied in a clinical scenario. Therefore, several therapies like using cells (NSCs, PSCs, etc.), biomaterials (scaffolds, hydrogel, etc.), and biomolecules (GFs, exosomes, etc.) are combined to reach their full potential. Combinational strategies can provide a cell‐friendly environment that simulate the niche of a healthy spinal cord. Either scaffolds/biomaterial‐based strategies, cell‐based strategies, or biomolecules‐based strategies, they are all targeted with one main aim: to facilitate the proliferation, differentiation, and migration of endogenous and exogenous cells at the injured sites.

Last but not the least, to reproduce the same effects of SCI regeneration in animal models and translate them into clinical use, three main challenges should be overcome^136^: the maintenance and effective expansion of stem cells, reliable protocols to provide leading cues for the differentiation of stem cell, and protection of cargos (cells, biomolecules, etc.) in the delivery system from mechanical stress and chemical invasion. In micro, overcoming these challenges can ensure the survival and vitality of endogenous and exogenous cells. In macro, insurance of vitality of cells can mitigate various syndromes of SCI,^34^, ^137^ like edema, hemorrhage, formation of cystic cavities, and ischemia. A deeper understanding of the mechanisms of how endogenous and exogenous cells facilitate functional recovery, as well as the acknowledgment of challenges of applying these therapies in clinical use and effectively constructing the spinal cord model, will be of huge importance to achieving successful SCI surgeries.

In conclusion, the successful functional regeneration of the injured sites in the spinal cord is an extremely tricky process, and the outgrowth of axons and effective connection between regenerated neurons and host neurons can be regarded as the successful combination of physical, biochemical cues. Future efforts and investigations should be made on discovering the optimal strategy for the functional regeneration of SCI and setting standards for the clinical use of bioengineering treatments. To be specific, the construction of scaffolds with precise and efficient delivery of therapeutic agents and various leading cues such as geometrical and electrical ones should be emphasized. Besides, seeking novel cells, cell‐derived biomolecules or their combinations which can support local cell proliferation and migration also deserve more attention. We believe that this comprehensive review will help accelerate the clinical transformation of SCI regeneration strategies from animal models to practical use in hospitals.

## AUTHOR CONTRIBUTIONS

Yingbo Shen, Hongcheng Gu and David A. Posner conceived the idea. Yingbo Shen wrote the manuscript with support from Xinyue Cao and Minhui Lu. Hongcheng Gu, Minli Li, and David A. Posner supervised the project and revised the manuscript. All authors discussed the results and contributed to the final manuscript.

## CONFLICT OF INTEREST

The authors declare no conflict of interest.
